# Variation in phenotype, genotype, and somatic diversity among asexual *Schmidtea mediterranea* planarians

**DOI:** 10.1016/j.isci.2025.113035

**Published:** 2025-07-01

**Authors:** Simon Kershenbaum, Danielle Ireland, Ziad Sabry, Christina Rabeler, Vir Shetty, Aziz Aboobaker, Eva-Maria S. Collins

**Affiliations:** 1 Department of Biology, University of Oxford, Oxford OX1 3SZ, UK; 2 Department of Biology, Swarthmore College, Swarthmore, PA 19081, USA; 3 Department of Neuroscience, Perelman School of Medicine, University of Pennsylvania, Philadelphia, PA 19104, USA

**Keywords:** natural sciences, biological sciences, genetics, phylogenetics, phenotyping, genotyping

## Abstract

Most multicellular life reproduces sexually, utilizing a single-celled stage that acts as a genetic bottleneck. This bottleneck limits the evolution of selfish cell adaptations by ensuring all cells descend from a single progenitor. We investigated an obligately asexual strain of *Schmidtea mediterranea* planarians that reproduces by self-bisection and lacks a single-cell bottleneck. Using 2.5 yearlong data on planarian reproductive behavior combined with genotyping, we revealed two previously undescribed genetically distinct substrains within the CIW4 strain. One substrain showed reduced fitness, which correlated with substantial losses of heterozygosity and increased somatic diversity. By genotyping consecutive head offspring over multiple generations, we found that only ∼9% of potential *de novo* mutations were transmitted to the next generation via the tail, suggesting that fission acts as a genetic bottleneck. Our study uncovers significant diversity in a fissiparous animal and proposes how somatic diversity can be controlled in the absence of a single-cell bottleneck.

## Highlights

• The asexual planarian strain CIW4 consists of two genetically distinct substrains
• The substrain with lower fitness shows evidence of increased somatic diversity
• Stem cell diversity is segregated across offspring during self-bisection
• Self-bisection may act as a genetic bottleneck in fissiparous planarians

## Graphical abstract

**Figure:**
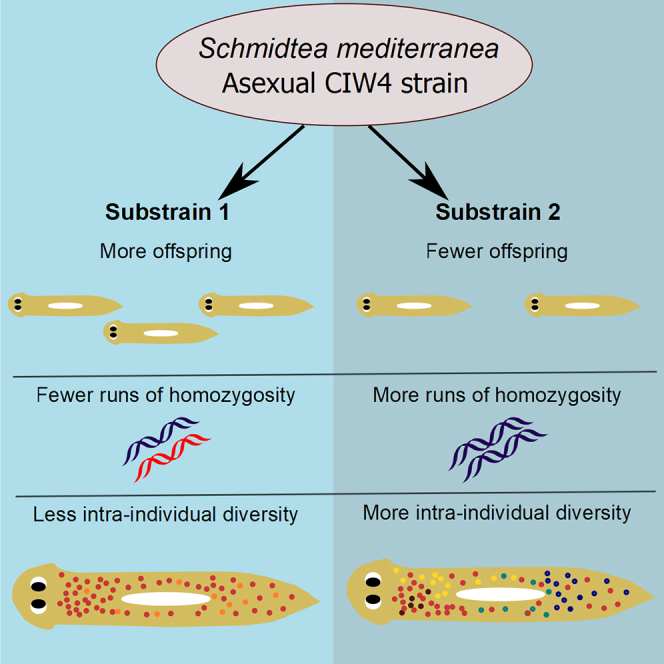

## Introduction

Sexual reproduction via a zygotic single-cell, genetic bottleneck (Table 1) evolved independently in all transitions to obligate multicellularity, suggesting that sex and frequent single cell bottlenecks are important for multicellular life.^1^^,^^2^ Some species lost the capacity to reproduce sexually and instead reproduce asexually either by parthenogenesis^3^^,^^4^ or by somatic reproduction via multicellular propagules (Table 1, e.g., fission, fragmentation, budding) that does not involve a single-cell bottleneck.^5^^,^^6^^,^^7^^,^^8^^,^^9^^,^^10^^,^^11^^,^^12^ Asexuality has short term benefits but is thought to be disadvantageous in the long term, which may explain the scarcity of ancient asexual species.^6^ Due to several different processes, asexual populations are limited in their ability to adapt to environmental change and evolutionary arms races.^13^^,^^14^^,^^15^^,^^16^ First, asexual populations cannot combine beneficial alleles from multiple individuals into a single offspring; thus beneficial mutations that arise in separate individuals must eliminate each other to reach fixation, a challenge known as clonal interference.^17^ Second, due to the tight linkage of alleles in asexual populations, selection functionally acts on the whole genome, rather than on individual alleles. This ultimately increases the impact of genetic drift and reduces the efficacy of selection in a process known as the Hill-Robertson effect.^18^^,^^19^ Third, due to the absence of sexual recombination, once drift removes the least mutated genotype in an asexual population, it cannot be easily restored, resulting in the progressive accumulation of deleterious mutations (Muller’s ratchet).^20^^,^^21^^,^^22^ However, it has been proposed that other forms of recombination leading to losses of heterozygosity may allow asexual lineages to purge deleterious mutations from the population.^23^^,^^24^^,^^25^ Overall, both parthenogenic and fissiparous species are limited in their adaptive potential compared to sexual species and need to acquire mechanisms to bypass these limitations to persist over evolutionary time.

**Table 1:** Definitions of major terms used in this study

Term	Definition
Genetic bottleneck	Condition where genetic variability is decreased between generations. For example, in sexual reproduction, a single-cell bottleneck exists wherein all cells arise from the single cell zygote.
Somatic reproduction	Asexual reproduction wherein multiple somatic cells form the offspring. Examples include binary division, fission, fragmentation, budding and vegetative propagation. For simplicity, we refer to organisms that undergo these types of reproduction as fissiparous.
Somatic diversity	Presence of cell lineages with different genetic identities in the soma/body of an individual; genetic mosaicism
Neoblasts	Planarian adult stem cells
Clonal	Product of mitotic division
Planarian lineage	Lineage of closely related planarians originating from a single founder
Substrain	Genetically distinct subpopulations within an existing strain
Allelic imbalance	A condition in which the observed ratio of alleles deviates significantly from the expected ratio. In the absence of somatic diversity, variant allele frequency is expected to be 0.5 in diploid organisms (heterozygous).

While the evolutionary implications of asexuality have been well studied in parthenogenic animals, they are not well understood in fissiparous species which are subject to additional challenges due to the lack of periodic single-cell bottlenecks.^12^ The resulting multicellular propagules, either as small body fragments or as small, fully formed buds, may contain multiple cell lineages, each with unique mutations acquired during the parent’s lifetime.^12^^,^^26^^,^^27^ This somatic diversity (Table 1) is subject to selection, which may select for selfish cells that contain mutations allowing them to outcompete their neighbors, such as higher proliferation rates, reduced differentiation, or skewed contribution to the germline.^12^

Various strategies can be employed to limit the emergence and persistence of selfish cell lineages in the absence of a single-cell bottleneck. For instance, some long-lived organisms exhibit reduced somatic mutation rates, thereby decreasing the likelihood of deleterious or parasitic variants emerging.^28^ Additionally, aggregative multicellular species often restrict cooperation to closely related individuals as a means of limiting somatic parasitism.^29^ Despite these mechanisms, selfish cell adaptations have been shown to occur in aggregative multicellular species including the ascidian *Botryllus schlosseri*, the fungi *Neurospora crassa*, and the facultatively multicellular *Dictyostelium discoideum*.^29^^,^^30^^,^^31^ However, little is known about the impact of reproduction via non-aggregative multicellular propagules (fission/budding/fragmentation) on population diversity, somatic diversity, and selfish cell evolution.

**Figure 1:**
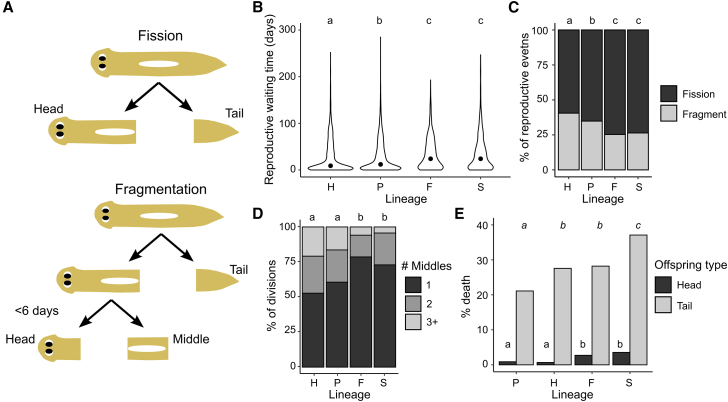
Planarian lineages show different levels of fitness (A) Schematic showing the different modes of asexual reproduction in *S. mediterranea*. In binary fission, two offspring—a head and tail—are created and then regenerate. In fragmentation, the head offspring divides again before regeneration is complete (<6 days) to create an additional “middle” offspring in addition to the original tail offspring. The head offspring can repeat this cycle multiple times before it fully regenerates, with larger worms being more likely to produce more offspring.^37^^,^^41^ We refer to each bisection event as a “division”, while the sum of all divisions comprising either a fission (one division) or fragmentation (>one division before regeneration is complete) is referred to as a “reproductive event”. (B) Violin plots of reproductive waiting time (time from birth to next division) for each lineage (Hans (H), Peter (P), Fritz (F), and Sophie (S)). Lineages are listed in the order of increasing median reproductive waiting time (dot). Groups with the same letter are not statistically different, groups with different lower-case letters are statistically significant (*p* < 0.01, Kruskal-Wallis omnibus test followed Dunn’s posthoc test with Bonferroni *p* value adjustment; *N*_H_ = 2,666, *N*_P_ = 3,138, *N*_F_ = 1,249, *N*_S_ = 953 biological replicates). (C) Percent of reproductive events that were fissions or fragmentations for each lineage. Groups with the same letter are not statistically different, groups with different lower-case letters are statistically significant (*p* < 0.01, Fisher’s exact test with Bonferroni *p* value adjustment; *N*_H_ = 1,552, *N*_P_ = 1,997, *N*_F_ = 945, *N*_S_ = 701 biological replicates). (D) Percent of fragmentations that produced different numbers of middle offspring for each lineage. For visualization, the creation of 3 or more middle pieces are consolidated into 3+. Groups with the same letter are not statistically different, groups with different lower-case letters are statistically significant (*p* < 0.05, pairwise comparisons of the estimated marginal means of a Poisson generalized linear model, with Bonferroni *p* value adjustment; *N*_H_: 629; *N*_P_: 696; *N*_F_: 239; *N*_S_: 185 biological replicates). (E) Percent death in each lineage for head and tail offspring. Percent is out of the total number of worms of that offspring type that died or divided. Lineages are listed in the order of increasing percent tail death. Different lower-case letters indicate statistically significant groups with separate comparisons across head (non-italicized) or tail (italicized) offspring (*p* < 0.05, Fisher’s exact test with Bonferroni *p* value adjustment; Heads: *N*_P_ = 2,389, *N*_H_ = 2,094, *N*_F_ = 847, *N*_S_ = 705; Tails: *N*_P_ = 976, *N*_H_ = 809, *N*_F_ = 592, *N*_S_ = 434 biological replicates).

To better understand the unique challenges associated with fissiparous reproduction, we ask how diversity is generated and partitioned in the asexual strain of *Schmidtea mediterranea* (CIW4), a freshwater planarian that reproduces solely by transverse self-bisection.^8^^,^^32^ Bisection occurs by elongating the body until it rips into two pieces, resulting in a large anterior offspring (head), and a smaller posterior offspring (tail),^33^^,^^34^ which subsequently regenerate over the course of 1–2 weeks to produce two fully functional planarians.^35^^,^^36^ Asexual *S. mediterranea* uses two modes of reproduction (Figure 1A): “Fission” produces one smaller tail offspring and a large head offspring^33^^,^^34^ whereas “fragmentation” consists of rapid, consecutive rounds of bisection within five days of each other, resulting in the production of one tail, one head, and one or more “middle” offspring that lack both head and tail.^37^ Successful reproduction relies on adult pluripotent stem cells called neoblasts (Table 1), which are the only cycling cells in the planarian body, and act to replenish tissues both during regeneration and homeostasis.^38^^,^^39^ As individual neoblasts accumulate mutations, somatic diversity increases. In the absence of a single-cell bottleneck to reset genetic homogeneity, somatic diversity can accumulate over generations. For example, asexual *Dugesia subtentaculata* planarians exhibit higher somatic diversity compared to their obligately sexual counterparts.^7^ However, it is unclear how increased somatic diversity impacts fitness and evolution in fissiparous animals. For example, while high levels of somatic diversity may drive genetic conflict within the body,^12^ it may also provide benefits by increasing genetic diversity and reducing the impact of genetic drift.^7^^,^^11^^,^^23^ Thus, more research is needed to understand how levels of somatic diversity relate to fitness in fissiparous animals. Asexual S. *mediterranea* evolved from a sexual ancestor ∼600,000 years ago and contains a translocation event between chromosomes 1 and 3,^8^^,^^40^ implying that they have been reproducing asexually without a single-cell bottleneck for potentially millions of generations. This makes them an ideal model to study the evolutionary implications of asexual reproduction in the absence of a single-cell bottleneck.

Our previous work has shown that despite the clonal (Table 1) nature of asexual *S. mediterranea*, there is variation in regeneration and reproductive dynamics between individuals that cannot be fully explained by offspring size (inherited biomass), offspring identity (head/middle/tail) and generational history (parent’s identity and mode of reproduction (fission/fragmentation)).^37^^,^^41^^,^^42^^,^^43^ Because significant somatic diversity has been shown in some fissiparous planarians,^5^^,^^12^ we speculated that phenotypic differences between offspring may be driven in part by genetic differences in their inherited neoblasts.^41^ Here, we aim to assess the diversity found within and between individual planarians and whether genetic differences between neoblasts underlie phenotypic diversity in asexual planarians. We utilized longitudinal data on the reproductive behavior and mortality rates from thousands of individual planarians from different lineages (Figure S1A, Table 1). Each lineage consisted of descendants originating from one respective asexual *S. mediterranea* founder via asexual reproduction events,^37^^,^^41^^,^^42^^,^^43^^,^^44^ and thus each lineage consists of closely related individuals. We combined this population data with genotyping of a subset of worms from each lineage and found that the four lineages could be separated into two distinct subpopulations with different reproductive fitness, patterns of homozygosity, and somatic diversity. These results show that the implicit assumption that the asexual CIW4 *S. mediterranea* strain represents a homogeneous population is incorrect. Moreover, by sequencing consecutive head offspring over multiple generations, we provide an estimate of the per generation mutation rate. We find evidence that fission can act as a genetic bottleneck by segregating genetically diverse neoblasts into different offspring. We suggest a model by which fission/self-bisection, acting as a genetic bottleneck, provides a mechanism for sustaining population diversity in asexual multicellular animals, while limiting accumulation of intra-individual somatic diversity. It has been previously shown that different species of asexual planarians, both obligate and facultative, reproduce at different rates with different offspring number and relative sizes, which can be explained by differences in the biomechanics of how they self-bisect.^34^^,^^42^ Therefore, further understanding how fission acts as a genetic bottleneck is needed to understand how these species-dependent reproductive traits, such as offspring number and size asymmetry of offspring may impact genetic diversity within individuals and populations. Taken together, our findings reveal unexpected diversity in clonal planarian populations, illustrating how even in the absence of meiosis, clonal animals can generate significant phenotypic and genetic variation.

## Results

### Differences in fitness between clonal planarian populations

The basis of this work is a unique multi-year long dataset tracking the reproductive behavior of thousands of individual planarians belonging to different lineages, which has been previously described^37^^,^^41^^,^^42^^,^^43^^,^^44^ (Dataset S1). Each lineage originated from, and is named after, a single planarian founder ((Peter (P), Hans (H), Fritz (F), and Sophie (S)), arbitrarily chosen from an established lab culture of asexual (CIW4) *S. mediterranea* planarians, which were allowed to reproduce naturally (Figure S1A). All resulting offspring over the next 2.5 years were cultured in individual petri dishes and their behavior (reproduction, death) was tracked with a custom barcode system.^44^

Although asexual *S. mediterranea* reproduce clonally, we have previously found variability in reproduction dynamics between individuals using an aggregated version of this dataset that could not be explained by known determinants, such as size and identity (head versus tail offspring).^37^^,^^41^^,^^42^^,^^43^^,^^44^ Thus, we first asked whether any systematic phenotypic differences could be found between the lineages of closely related individuals. We found a significant difference in population-level dynamics across the planarian lineages. Both P and H lineages had significantly lower median reproductive waiting times than the F and S lineages (*p* < 0.001, Kruskal-Wallis omnibus test followed Dunn’s posthoc test with Bonferroni *p* value adjustment; Figure 1B). P/H worms also displayed significantly more fragmentations (*p* < 0.01, Fisher’s exact test with Bonferroni *p* value adjustment; Figure 1C) and produced on average more middle offspring per fragmentation than the F/S lineages (*p* < 0.05, pairwise comparisons of the estimated marginal means of a Poisson generalized linear model, with Bonferroni *p* value adjustment; Figure 1D), implying that the P/H lineages were more successful reproducers. Moreover, the P/H offspring were more likely to survive and reproduce again (Figure 1E). P/H head offspring had significantly higher survival rates than F/S head offspring (*p* < 0.01, Fisher’s exact test with Bonferroni *p* value adjustment). This trend was also present in tail offspring (*p* < 0.05, Fisher’s exact test with Bonferroni *p* value adjustment), which we have previously shown have much higher probabilities of dying,^42^ though differences between H and F were not significant. These data suggest that the P and H lineages have increased fitness (more frequent divisions producing more offspring and increased survivability) than the F and S lineages.

### Genotype analysis of the planarian lineages reveals two distinct asexual substrains

**Figure 2:**
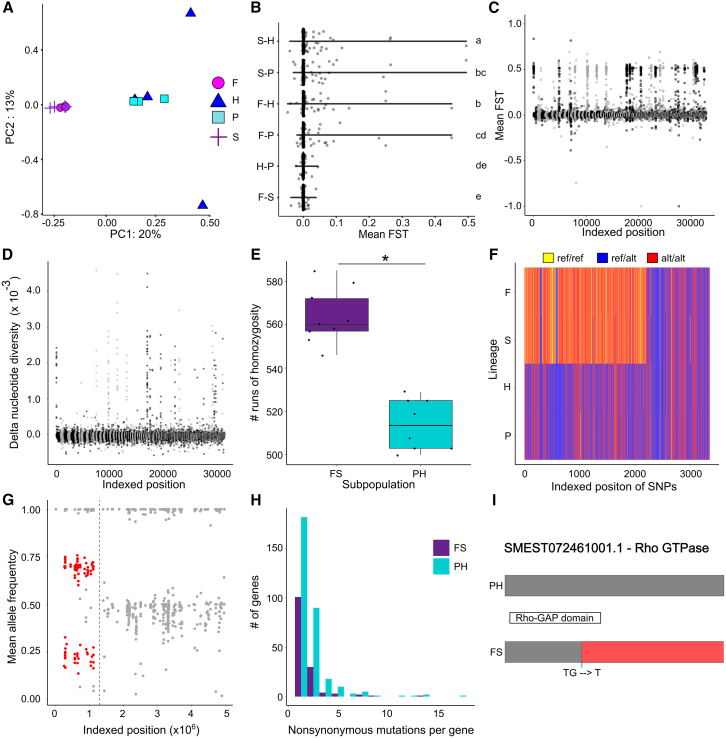
PH and FS represent two distinct substrains (A) PCA showing that individuals from the F and S lineages segregate from individuals from the P and H lineages. Only genotypes with GQ > 20 across all samples were used for this analysis to remove bias due to depth differences (Figure S2). (B) Mean pairwise fixation index scores (FST) per scaffold (individual dots) between different lineages. Between cluster comparisons show scaffolds with high positive FST scores, suggesting that FS and PH represent distinct subpopulations. Groups with the same letter are not statistically different, groups with different lower-case letters are statistically significant (*p* < 0.001, Wilcoxon rank sum with Bonferroni *p* value adjustment, *N* = 432). (C) FST scores across the genome between the FS and PH subpopulations show peaks of genetic distance. Included scaffolds are colored in alternating black and gray for ease of visualization. (D) Difference in nucleotide diversity scores within the PH and FS subpopulations (PH_diversity_ − FS_diversity_). PH has peaks of diversity that are absent from FS (but not vice versa). Included scaffolds are colored in alternating black and gray for ease of visualization. (E) There are more runs of homozygosity in FS compared to PH (*p* < 0.001, Wilcoxon rank sum; *N*_FS_ = 9, *N*_PH_ = 8, *W* = 72). (F) Visualization of an FS-specific run of homozygosity in scaffold dd_Smes_g4_1 (the first 2,000,000 bps). Each line represents a variant that is called in all samples, and their position is indexed based on the position in the scaffold. (G) Mean allele frequencies within a portion of scaffold dd_Smes_g4_1 using various published BioProjects (see Figure S4). This shows inconsistency in zygosity in the region of the FS-specific run of homozygosity, suggesting that FS and PH subpopulations are mixed in CIW4 populations across the world and represent distinct substrains. Variants within the region of the FS-specific run of homozygosity in dd_Smes_g4_1 are in red, and the dashed line indicates the end of the run of homozygosity. (H) Number of genes containing different numbers of subpopulation-specific nonsynonymous mutations. (I) Schematic of the FS-specific frameshift deletion found in the Rho GTPase coding sequence. The regions impacted by the frameshift are in red.

We asked whether we could explain the observed differences in fitness between the lineages from differences in their genotypes. We performed genomic sequencing on 4–5 individuals from each of the four planarian lineages (Figure S1B). Principal component analysis (PCA) on the high-quality genotypes of the sequenced individuals from each lineage showed that individuals from the P and H lineages segregated from those from the F and S lineages, suggesting that they are distinct genetic subpopulations (Figure 2A). Interestingly, while the F and S individuals were closely clustered together, individuals from the H and P lineages were more spread out, potentially suggesting greater inter-individual variability. Pairwise fixation index scores (FST) confirmed that the P/H and F/S lineages represent distinct subpopulations within our dataset. Despite all FST scores being very low, demonstrating the lineages are very genetically similar, clear population structure was present. Within group FST scores (e.g., F-S or P-H) were centered around 0, indicating the lineages are part of the same population, whereas across group comparisons of F/S to P/H had positively skewed FST scores (*p* < 0.001, Wilcoxon rank sum with Bonferroni *p* value adjustment; Figure 2B), indicating that these are distinct subpopulations. These across group differences seemed to be driven by a small number of scaffolds that showed high positive FST scores. In agreement with this result, when comparing FST scores between the FS (F and S) and PH (P and H) subpopulations, we found that genetic differences are mostly found at discrete peaks across the scaffolds (Figure 2C), rather than spread equally across the genome. Thus, the FS and PH lineages comprise distinct genetic subpopulations, which correlate with the observed differences in fitness and contain distinct peaks of divergence across the genome (Figure 1).

To better understand how the PH and FS subpopulations were genomically distinct, we compared nucleotide diversity (mean number of nucleotide differences per site) across the genomes of both groups. We found that PH had peaks of nucleotide diversity that were absent from FS (Figure 2D), suggesting that in certain regions of the genome, the PH subpopulation contains noticeably higher diversity than FS, while the reverse pattern was largely absent. Notably, this high diversity in the PH lineages was detected despite the lower sequencing depth of PH individuals (Figure S2). One way in which peaks of diversity can arise between closely related strains are losses of heterozygosity. In asexual populations, losses of heterozygosity can occur due to hemizygous deletions, gene conversion, and other forms of mitotic recombination.^23^^,^^24^^,^^25^ To determine if the reduced diversity in FS is due to the losses of heterozygosity, we compared patterns of homozygosity across the genomes of individuals from each lineage. We found that all lineages contained runs of homozygosity (long stretches of homozygous regions, see STAR Methods, Dataset S2) in all four chromosomes (Figure S3A), and that the FS lineages contained significantly more runs of homozygosity than PH (*p* < 0.001, Wilcoxon rank sum; W = 72; Figure 2E). Moreover, we found that while PH contained only 31 genes within specific runs of homozygosity, FS had 474 genes within its specific runs of homozygosity (Figure S3B). These runs of homozygosity cannot be explained by large scale deletions since there is only a small drop in depth within FS-specific runs of homozygosity (*p* = 0.005; Wilcoxon signed rank; V = 99; Figure S3C). Additionally, they are unlikely to be purely the result of GC-biased gene conversion since these runs of homozygosity are AT-rich compared to the rest of the genome (*p* < 0.01; Wilcoxon signed rank; V = 127; Figure S3D), and the variants in FS-specific runs of homozygosity are not GC-biased (*p* = 0.1; Wilcoxon signed rank; V = 14; Figure S3E). One of the clearest examples of an FS-specific run of homozygosity can be found in the dd_Smes_g4_1 scaffold and is over 1.3 Mb in length (Figure 2F).

To determine whether these distinct subpopulations could also be found in CIW4 cultures from other labs, we examined the region containing this FS-specific run of homozygosity in previously published asexual *S. mediterranea* variant data (Figures 2G and S4; BioProject: PRJNA298875, PRJNA293934, PRJNA283132).^45^^,^^46^ We found atypical allele frequencies in this region in the various external datasets, with allele frequencies values between 0.5 and 1. Since pure populations of PH or FS should exhibit allele frequencies of 1 or 0.5, respectively, an intermediate value suggests that the two subpopulations are mixed within these different CIW4 populations. Interestingly, this pattern was absent from the independently generated asexual *S. mediterranea* strain BCN10,^47^ although only data from a single BioProject was used (BioProject: PRJNA222859).^48^ Thus, we have identified two distinct substrains of the asexual CIW4 strain of *S. mediterranea*, with significant genetic and phenotypic differences. Because these differences are found within the CIW4 asexual strain, we refer to the subpopulations as substrains, rather than separate strains.

To understand how the genetic differences between the substrains may underlie their phenotypic differences, we assessed the variants that were specific to each substrain (Dataset S3). Most variants were non-specific and found in at least one FS and PH sample (92.2%); however, this still left 3,684 FS-specific SNPs and 10,782 PH-specific SNPs. We found 142 genes with FS-specific nonsynonymous mutations, of which 41 genes contained more than one FS-specific nonsynonymous mutation, and 314 genes with PH-specific nonsynonymous mutations, of which 132 genes contained more than one PH-specific nonsynonymous mutation (Figure 2H, Dataset S6). Notably, 4,262 of the PH-specific SNPs were found in regions corresponding to FS-specific runs of homozygosity, suggesting these PH-specific SNPs were lost in FS because of the loss of heterozygosity. In contrast, only 4 FS-specific SNPs were found in regions containing PH-specific runs of homozygosity. Moreover, we identified all substrain-specific indels (Table S3) and found a single FS-specific TG → T frameshift deletion in SMEST072461001.1, which encodes a Rho GTPase (Figure 2I). Rho GTPases are important molecular switches that are involved in a myriad of cellular processes including cell division and migration.^49^ Notably this deletion was in the region encoding the active Rho-GAP domain and would likely produce a nonfunctional or malfunctioning protein. This frameshift deletion was heterozygous in all FS individuals (9/9), thus, whether it has a recessive or dominant effect is unknown. We also found ten genes with PH-specific frameshift indels (Table S3), with only three containing the frameshift in a region that would impact domain structure. These three genes blasted to a piggyBac related gene, a patched-like protein, and a hypothetical protein in *Schistosoma haematobium*. All frameshift mutations in these three genes were heterozygous (8/8). Given the increased fitness of the PH substrain over the FS substrain, it is unclear whether any of these frameshifts are functionally deleterious. Taken together, although the FS substrain harbors fewer nonsynonymous and frameshift mutations, it contains over ten times more genes within substrain-specific runs of homozygosity, potentially exposing these genes to harmful recessive mutations which, alongside a deleterious frameshift mutation, may contribute to its reduced fitness.

### Low fitness in the FS substrain correlated with high somatic diversity

**Figure 3:**
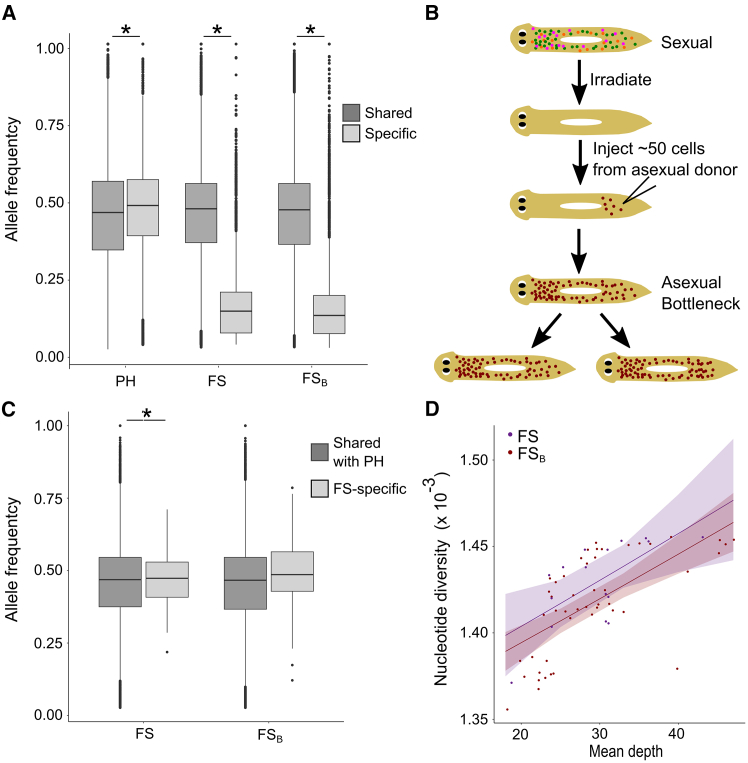
Intra-organismal diversity in the FS substrain is not decreased in the presence of a ∼50 cell bottleneck (A) Comparison of allele frequency of variants that are either shared (found in at least one individual from both PH and FS) or specific (found in at least one individual from either PH or FS but absent in the other substrain). Specific variants in the bottleneck (FS_B_) families are FS-specific variants also found in FS_B_ individuals. ∗Significant differences between shared and specific variants in the same group (*p* < 0.001, Wilcoxon signed rank; *N*_PH-shared_ = 945,253, *N*_PH-specific_ = 49,339, *N*_FS-shared_ = 1,314,914, *N*_FS-specific_ = 5,393, *N*_FSB-shared_ = 2,094,813, *N*_FSB-specific_ = 9,900; Mean_PH-shared_ = 0.44, Mean_PH-specific_ = 0.46, Mean_FS-shared_ = 0.44, Mean_FS-specific_ = 0.16, Mean_FSB-shared_ = 0.44, Mean_FSB-specific_ = 0.15). (B) Schematic showing creation of bottleneck individuals. Sexual planarians containing different populations of stem cells (colored dots) were lethally irradiated to remove all stem cells and were subsequently rescued by injection of approximately 50 cells (red dots) from a single asexual CIW4 donor. Thus, all cells in the two rescued bottleneck worms (B1 and B2) arose from those original 50 cells (of which only a subset is likely to be totipotent neoblasts). Each of the rescued worms was then used as a founder for a new planarian lineage. (C) Considering only variants that were supported by all FS and bottleneck (FS_B_) individuals, comparison of the allele frequency of mutations shared with PH and mutations specific to the FS-substrain. ∗Significant difference between shared and specific variants in the same group (*p*_FS_ = 0.589, *p*_FSB_ < 0.001; Wilcoxon rank sum; *W*_FS_ = 11,934,818, *W*_FSB_ = 36,931,454, *N*_FS-shared_ = 214,569, *N*_FS-specific_ = 108, *N*_FSB-shared_ = 357,615, *N*_FSB-specific_ = 180; Mean_FS-shared_ = 0.45, Mean_FS-specific_ = 0.47, Mean_FSB-shared_ = 0.45, Mean_FSB-specific_ = 0.49). (D) Plot of mean nucleotide diversity per scaffold as a function of sample depth in FS and FS_B_ lineages. Shaded regions represent 95% confidence intervals. A quasibinomial generalized linear model found that bottlenecking is not a significant predictor for nucleotide diversity (*p* = 0.807; Figure 1, Figure 2, Figure 3, Figure 4, Figure 5C).

Genetic diversity may also be present within the body of individuals, creating somatic diversity or genetic mosaicism. In the absence of a single cell bottleneck, as in asexual *S. mediterranea*, this genetic diversity may be heritable and could contribute to the overall diversity in the population, depending on whether the mutations are in totipotent neoblasts that will be passed on to the next generation.^5^^,^^12^^,^^50^ Thus, we assessed whether asexual *S. mediterranea* show evidence of significant somatic diversity and whether this differs between the asexual substrains. In the absence of somatic diversity, it is expected that alternative alleles at biallelic sites would be supported by approximately 50% of sequencing reads (allele frequency = 0.5), suggesting that all cells are heterozygous. Deviation from this value (allelic imbalance, Table 1) suggests the presence of alleles that are not shared across all cells in the body (some cells are heterozygous and some are homozygous reference), indicating the presence of somatic diversity. When assessing all alternative variants, FS and PH show similar variant allele frequencies distributions with similar means (mean_PH_ = 0.438, mean_FS_ = 0.440, Figure S5). The distributions of allele frequencies are driven by the variants shared between the substrains, which likely arose prior to the strains diverging. These shared variants represent the vast majority of variants and had a mean allele frequency close to 0.5 in both substrains, suggesting that they are found in all cells in the body (Figure 3A). In both substrains, there are a small subset of shared variants with low allele frequencies, which are likely due to SNPs in repetitive regions. However, substrain-specific variants, which likely arose after the two substrains diverged, showed differing patterns. Variants specific to PH had an average allele frequency close to 0.5. However, FS-specific variants showed a significantly lower allele frequency (*p* < 0.001, Mean_FS-specific_ = 0.16, Wilcoxon signed rank), which suggests higher levels of somatic diversity. While these patterns could potentially be explained by polyploidy, widespread duplications, or strain-specific sample contamination, they were all rejected as potential explanations for the allelic imbalance in FS-specific variants (Figure S6). Specifically, we evaluated the karyotypes of 10 planarians derived from the FS-substrain (*n* ≥ 10 chromosome spreads each) and found no evidence of polyploidy (Figure S6A). We also found no differences in mapping rates between PH and FS libraries (*p* > 0.05 for all comparisons, Wilcoxon rank sum exact test with Bonferroni *p* value adjustment; Figure S6B), suggesting that contamination is not likely to explain these differences. Finally, local duplications were rejected as an explanation for the low somatic allele frequency of FS-specific variants since these specific variants showed lower sequencing depth than shared variants (*p* < 0.001; Wilcoxon rank sum; *W* = 303,804,886; Figure S6C).

### Intra-organismal diversity is not affected by a 50-cell bottleneck

Given the high levels of somatic diversity observed in the FS substrain we asked whether an experimentally induced cellular bottleneck would reduce the observed intra-organismal diversity. We produced two new lineages from experimentally bottlenecked *S. mediterranea* individuals. Two bottleneck individuals derived from a small number of neoblasts from the same CIW4 donor were generated using a previously described technique^38^^,^^51^ (Figure 3B, see STAR Methods). We originally sought to create single cell bottlenecks as previously described,^38^^,^^51^ but were unable to obtain successful rescues after a few hundred attempts due to the extremely low success rate of this procedure.^38^^,^^52^ Therefore, we injected ∼50 cells, of which only a subset are likely to be pluripotent neoblasts. Out of 180 injected planarians, only two survived and regenerated and were named bottleneck 1 (B1) and bottleneck 2 (B2). B1 and B2 were used as founders to grow two planarian lineages via asexual reproduction over a period of 2.5 years (STAR Methods). Despite being generated several years after the termination of the original lineages, B1 and B2 were found to be derived from the same CIW4 substrain as the F and S lineages (Figure S7). Thus, we refer to them as FS_B_ and made comparisons to the F and S lineages. Genomic sequencing of 7–8 individuals from each bottleneck lineage was used to evaluate whether a cellular bottleneck would affect somatic diversity.

We found that despite the ∼50 cell bottleneck, FS-specific variants (found in at least one FS individual but no PH individuals) still showed an allelic imbalance indicative of high intra-organismal diversity in the bottleneck lineages (Mean_FSB-specific_ = 0.15; Figure 3A). However, FS-specific variants that were found in all F, S, and FS_B_ sequenced individuals (likely to be ancestral variants to the FS substrain) did not show an allelic imbalance (Mean_FSB-shared_ = 0.44; *p*_FS_ = 0.589, *p*_FSB_ < 0.001; Wilcoxon rank sum; *W*_FS_ = 11,934,818, *W*_FSB_ = 36,931,454, *N*_FS-shared_ = 214,569, *N*_FS-specific_ = 108, *N*_FSB-shared_ = 357,615; Figure 3C). Thus, although new variants in the FS substrain show an allelic imbalance and therefore are likely not found in all cells in the body, over time the variants become diploid heterozygous variants in all cells. Additionally, we found no difference in the per scaffold nucleotide diversity of the unperturbed FS lineages vs. the bottleneck lineages (Quasibinomial GLM; p_bottlenecking_ = 0.807, p_Mean sample depth_ < 0.001, p_interaction_ = 0.921, Figures 3D and S2C), and found no differences in heterozygosity between the groups (*p* = 1, Wilcoxon rank sum with Bonferroni *p* value adjustment, Figure S2D), suggesting that a ∼50 cell bottleneck is sufficient to capture the intra-organismal diversity present in the FS substrain.

We next asked whether the introduction of an experimental cellular bottleneck had phenotypic consequences on the resulting lineages. The bottleneck lineages had significantly longer reproductive waiting times (*p* < 0.001, Kruskal-Wallis omnibus test followed Dunn’s posthoc test with Bonferroni *p* value adjustment; Figure S8A) and fewer fragmentations than F/S (*p* < 0.001, Fisher’s exact test with Bonferroni *p* value adjustment; Figure S8B). However, there was no type-specific (unperturbed vs. bottleneck) effect on survival rate (Fisher’s exact test with Bonferroni *p* value adjustment; Figure S8C). Given that there is no difference in inter- or intra-organismal diversity between the FS and the bottleneck lineages (Figures 3 and S2D), we suspect one explanation for the reduced fitness in the bottleneck lineages may be a consequence of the trauma of the cellular bottleneck. To test this idea, we evaluated whether these differences in reproductive dynamics changed over the course of the 2.5 years of cultivation since the creation of the bottleneck founders. We found in all lineages (F, S, B1, and B2) relative birth month had a significant effect on reproductive waiting time (*p* < 0.01 for each lineage, Kruskal-Wallis test), as worms born later after the creation of their respective lineage tended to have shorter reproductive waiting times (Figure S8D). Moreover, while the reproductive waiting time distributions of the bottleneck lineages differed dramatically from those of the non-perturbed lineages in the beginning of the experiment, they became more similar for worms born later relative to the founding of their lineage (Figure S8D).

### Fission may act as a genetic bottleneck

**Figure 4:**
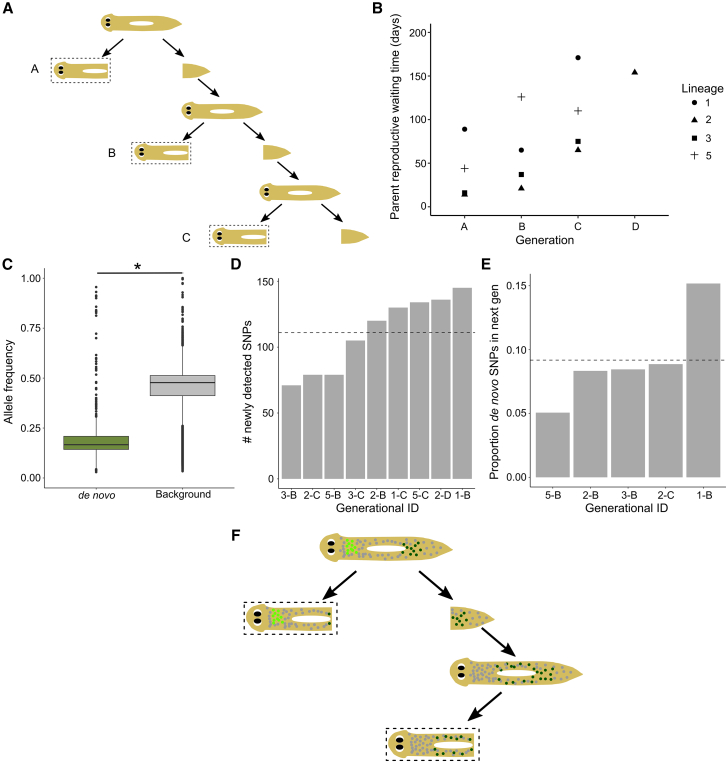
Mutation rate per generation (A) Schematic of experimental design. From a given founder planarian, consecutive head offspring produced by fission were collected and sequenced (dashed boxes) while the tail offspring was allowed to continue dividing. Each tail line consisted of 3–4 consecutive generations. (B) Plot of the parent reproductive waiting time per generation for each lineage to show the hundreds of days long time-scales needed for consecutive tail fissions. Generation of these lines was also complicated by the fact that tail offspring have a >20% death rate (Figures 1E and S8C). (C) *De novo* mutations have a lower allele frequency compared to established background mutations that are present in all individuals. ∗Statistically significant: *p* < 0.001; Wilcoxon rank sum; *W* = 11,461,506, *N*_denovo_ = 746, *N*_background_ = 135,855. (D) Number of newly detected SNPs in each library (see STAR Methods). Dotted line shows mean. (E) Proportion of *de novo* mutations that are present in the next generation. Dotted line shows mean. (F) Schematic showing how variants may (dark green) or may not (light green) be inherited by the tail (and thus would or would not be present in the next sequenced generation, respectively).

To understand how rapidly somatic genetic variation is generated, we assessed the number of new mutations detected per generation in the bottleneck lineages. To this end, we sequenced the consecutive head offspring stemming from the same tail offspring, which was allowed to undergo consecutive rounds of fission and regeneration (Figure 4A). Because tails have a high probability of dying (Figures 1E and S8C) and divide slowly (Figure S9B), generation of consecutive head lineages consisting of 3–4 head offspring took 128–325 days (Figure 4B). Established variants in the population, i.e., variants shared by both substrains, have a mean frequency of 0.433 in the sequenced head offspring, suggesting that they are mostly heterozygous variants found in all somatic cells, while newly detected variants had a frequency of only 0.2, suggesting that they are likely only found in a subset of cells (*p* < 0.001; Wilcoxon rank sum; *W* = 33,137,334; Figure 4C). Newly detected variants were defined as those found only in the focal individual and absent from all other tested libraries excluding their direct progeny (Figure S10A). By assessing the proportion of SNPs that are heterozygous in all the cells of the parent worm (allele frequency of 0.5) but absent from the offspring, we calculated a false negative SNP calling rate of 0.008 ± 0.005 (mean ± standard deviation; Figure S10B). This low false negative SNP calling rate suggests that newly detected mutations are highly unlikely to be due to false discovery in the other 35 libraries that were cross-referenced. There were on average 111 ± 28.4 (mean ± standard deviation) newly detected mutations per generation (Figure 4D), which represents a mutation rate of ∼3.75 × 10^−7^ per bp per generation (see STAR Methods). Due to the polyclonal nature of these planarians, it is possible that newly detected mutations do not represent *de novo* mutations but instead include mutations that have sufficiently increased in frequency since the last generation to be detected in bulk genome sequencing.

Of the newly detected mutations, only 9.2 ± 3.7% (mean ± standard deviation) were also detected in the next head generation stemming from the tail offspring (Figure 4E). This suggests that most new variants are either not present in the tail at the time of fission or are rapidly removed from the tail piece following fission, suggesting that fission may act as a genetic bottleneck (Figure 4F). An alternative explanation is that only ∼9% of all newly detected variants are true *de novo* mutations (verified by their presence in the next generation), and that most of the rest are simply false positives due to sequencing errors or other technical limitations. However, we did not find clear differences in the mutational patterns between newly detected mutations and the rest of the variants in our dataset, suggesting that they are unlikely to be driven by sequencing errors (Figure S11). When considering only newly detected mutations that are found again in the next generation, asexual *S. mediterranea* show a mutation rate of ∼3.31 × 10^−8^ per bp per generation. For both mutation rates, we only considered mutations within gene bodies. Moreover, while we did not identify any genomic regions with particularly high mutations rates (Figure S7E), it is possible that the mutation rate is not representative of the whole genome and may be dominated by mutations in highly mutable regions. Taken together, these data suggest that while new mutations arise rapidly in the soma, only a portion of these mutations are detected again in the next generation, suggesting that fission may act as a genetic bottleneck, partitioning somatic variation into separate individuals.

## Discussion

### Why are there two distinct substrains in a recent clonal population?

We found significant phenotypic and genetic variation that differentiates two previously undescribed substrains within the CIW4 strain of fissiparous *S. mediterranea*. The FS substrain is characterized by runs of homozygosity that are absent from the PH substrain. These differences in homozygosity can be driven by asexual mechanisms, such as automixis,^53^ GC-biased gene conversion,^54^^,^^55^ break induced replication,^56^ or other forms of mitotic recombination. However, such signatures could also be driven by inbreeding,^57^ raising the possibility of cryptic sex in asexual S. *mediterranea.* We believe that inbreeding is an unlikely explanation for these runs of homozygosity for two main reasons: (1) Since the establishment of the CIW4 line in 1999,^58^ this population has never been observed to reproduce sexually or develop gonads^8^^,^^59^^,^^60^; (2) Sexual S. *mediterranea* are cross-fertilizing hermaphrodites, meaning that even if an asexual individual sexualized, it would require another sexual individual to reproduce.^61^ While some asexual planarian species that lack chromosomal translocations can be sexualized experimentally, this has not been achieved in *S. mediterranea*.^62^^,^^63^^,^^64^ Thus, it is more likely that the differences in homozygosity between the two substrains are the result of large-scale mitotic recombination events. This is of particular interest since it suggests that relatively frequent, large-scale mitotic recombination events occur in asexual *S. mediterranea.* Such mitotic recombination events have been suggested to be important for purging deleterious mutations in asexual lineages.^23^^,^^24^

Additionally, we find that chromosome 1, which accounts for more than 40% of the *sexual S. mediterranea* genome,^65^ has the fewest bps within runs of homozygosity (Figure S3A). This is true for runs of homozygosity shared between the PH and FS substrains (likely to be ancestral), but not substrain-specific runs of homozygosity which likely arose recently (Figure S3A). The scarcity of ancestral homozygous regions on chromosome 1 in asexual *S. mediterranea* may reflect inheritance from a sexual ancestor that already exhibited recombination suppression on this chromosome, as observed in extant sexual populations from Sardinia and Corsica.^65^ We therefore propose that asexual *S. mediterranea* may have originated from a sexual lineage with pre-existing recombination suppression on chromosome 1.

### Potential genetic causes for differences in fitness between the substrains

We found that the PH substrain reproduced more rapidly, produced on average more offspring per reproductive event and had decreased mortality compared to the FS substrain. The reduced mortality in the PH substrain was surprising because it has previously been shown that fragmentation events that create >4 offspring (i.e., >3 middles) cause subsequent declines in offspring survival, including of the head.^37^ All middle offspring originate from the head piece in *S. mediterranea*. Thus, their creation causes a decrease in head offspring size while not affecting tail offspring size.^34^^,^^37^ The creation of more middle offspring lowers the fitness of the head, increasing its reproductive waiting time and probability of dying.^37^^,^^42^ Therefore, it is surprising that despite the known increased cost of multi-offspring fragmentations, the PH head offspring had a higher survival probability than the FS head offspring. This may be another indicator of the increased fitness of PH compared to FS.

There are several non-mutually exclusive possible reasons for the differences in fitness between the substrains. First, losses of heterozygosity in the FS substrain may have exposed the deleterious impact of recessive mutations. Reduction in fitness due to loss of heterozygosity has been shown in both sexual (inbreeding depression^66^) and asexual species^54^ and is likely to play some role in the reduced fitness of FS, since the FS substrain has >10× the number of genes impacted by long runs of homozygosity compared to the PH substrain. Second, FS-specific mutations may reduce the fitness of this substrain. For example, the FS-specific frameshift deletion in a Rho GTPase gene would likely dramatically impact its protein function. Rho GTPases are important in a number of key cellular processes including cell division and migration.^49^ However, it is unknown whether this heterozygous mutation would have a dominant or dose effect to impart a phenotypic change. It is also possible that the heterozygous PH-specific frameshift mutations cause negative effects but based on our data do not lower the fitness of PH compared to FS. Overall, we found more substrain-specific mutations in the PH substrain. Many PH-specific mutations were found in regions corresponding to FS-specific runs of homozygosity, implying that these PH-specific variants may have been lost in FS because of the loss of heterozygosity in these regions. It is also possible that the increased somatic diversity in the FS substrain led to difficulties in calling variants with a low allele frequency and thus the number of mutations in FS may be underestimated. Thus, PH-specific variants may be more easily detected due to their higher genetic homogeneity. Lastly, the somatic diversity in the FS substrain may also reduce organismal fitness due to selection for selfish cell adaptations in FS neoblasts. Such processes have been seen in aggregative multicellular organisms such as *Dictyostelium discoideum*, *Botryllus schlosseri*, and *Neurospora crassa*,^12^^,^^29^^,^^30^^,^^31^ but has not yet been described in obligately fissiparous species such as asexual *S. mediterranea*. While we do not assess the differences in selfish cell behavior between the FS and PH substrains, we suggest that it may contribute to the reduced fitness in the FS substrain and should be further examined.

Moreover, we found that the bottleneck lineages have reduced reproductive rates compared to the non-perturbed FS lineages, despite having the same levels of somatic diversity. In the original single cell rescue study,^38^ the authors followed the reproductive behavior of three rescued worms. They cut each rescued planarian around 100 days post-injection and only 1/3 had naturally divided within the first 150 days, thus similarly showing low reproductive rates within the first ∼200 days following rescue.^38^ In both the bottleneck and non-perturbed lineages, we found that reproductive waiting times were affected by how long the worms were cultured individually in petri dishes (see Figure S8D). Planarians born later relative to the creation of their respective lineage tended to have shorter reproductive waiting times, with the bottleneck lineages approaching the reproductive waiting time distributions of the unperturbed lineages over time. These data suggest that the experimental bottleneck may have caused trauma or other lasting effects that took several generations to overcome. One possibility is that deleterious mutations were enriched in the original selection of neoblasts that rescued the irradiated hosts and these were progressively purged over time. Unfortunately, we were unable to look at genetic trends over time as extraction of enough genomic DNA for sequencing requires sacrificing the whole animal, which would preclude its ability to continue populating the lineage. Thus, all sequenced individuals were from generation 10 or 11, once sufficient reproductive data had been obtained. Non-genetic effects could also have impacted the health and reproductive behavior of the bottleneck lineages. For example, long-lasting epigenetic responses could be triggered by cell dissociation, injection into a radiation-exposed soma, or incompatibility between asexual neoblasts within a sexual planarian host. These factors may have contributed to the transient reduction in fitness observed in the bottleneck individuals. However, ultimately the underlying mechanism behind these non-genetic effects on fitness is unknown and should be further examined to understand how rescue experiments impact planarian reproductive behavior in the short term.

### Bisection as a potential genetic bottleneck

In the absence of a single cell bottleneck, fissiparous planarians have been shown to accumulate high levels of somatic diversity.^7^^,^^50^ We found that even between two closely related substrains of asexual *S. mediterranea* planarians there are dramatic differences in somatic diversity, with only the FS substrain showing significant levels of somatic diversity. This difference in somatic diversity suggests that the FS substrain is not as efficient in generating genetic bottlenecks compared to its PH counterparts. The different reproductive dynamics of the two substrains may at least partially explain these differences as PH individuals on average divided more frequently and produced more offspring per reproductive event (more multi-offspring fragmentations). In addition to differences in reproductive dynamics, variation in somatic diversity could also be explained by differences in cell mixing or mutation rates between the substrains, which may be impacted by substrain-specific mutations such as the Rho GTPase frameshifting indel in the FS substrain.

Overall, we hypothesize that variation in somatic diversity between closely related asexual planarians is driven by differences in their reproductive dynamics. Since different asexual planarian species (both obligate and facultative) reproduce at different rates with variable offspring number and size,^34^^,^^42^ we expect to see variation in somatic diversity between asexual planarian species.

**Figure 5:**
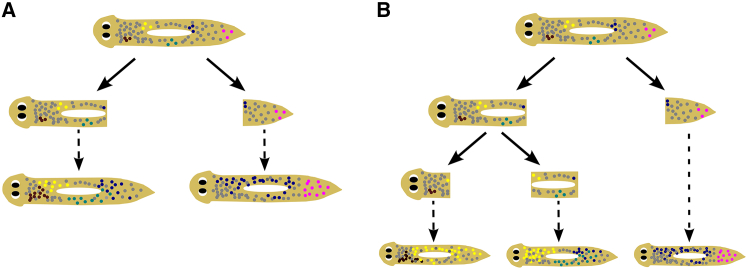
Proposed model of how different reproductive dynamics may impact somatic and population diversity (A and B) Within a given generation, new mutations (colored dots) originally arise in a single neoblast and are propagated to a small population of clones descending from that neoblast, resulting in somatic diversity. The diverse subpopulations of neoblasts are physically segregated across the offspring during self-bisection (solid arrows, A shows fission and B shows fragmentation), leading to the creation of diverse offspring following regeneration (dotted line). If more offspring are created, i.e., during fragmentation in (B), then the genetic diversity is split into smaller pieces and the resulting offspring have less somatic diversity within them, as is seen in the PH substrain. We hypothesize this may also lead to greater inter-individual heterogeneity within the population.

Figure 5 shows our proposed model of how different modes of reproduction can affect somatic diversity in fissiparous planarians. *De novo* mutations will originally arise within a single neoblast and then propagate to a small population of clonal cells. Previous studies have shown that neoblast migration is generally limited to local wound sites,^67^ implying that neoblast lineages may maintain spatial organization. Thus, because of this spatial segregation of diverse neoblast lineages, self-bisection would segregate genetic diversity among the offspring and limit the accumulation of somatic diversity within a given individual. The creation of multiple offspring during fragmentation would therefore further decrease somatic diversity compared to fission. In addition to decreasing intra-individual diversity, we hypothesize that segregation of diversity into more offspring (fragmentation) would increase population-level inter-individual diversity. However, we were unable to directly test this due to the reduced depth of the PH substrain sequenced samples (Figures S2A and S2B).

Our data support the hypothesis that self-bisection acts as a genetic bottleneck in asexual *S. mediterranea* and partitions diversity among offspring as we found that only ∼9% of newly detected mutations are transmitted to the next generation via the tail. Other potential mechanisms may explain the low heritability of somatic mutations in fissiparous planarians. For example, it is possible that only a limited subset of neoblasts contributes to the long-term genotype of a planarian. This could result from intense stem cell competition,^68^^,^^69^ whereby most neoblast lineages are lost over time, along with their lineage-specific mutations. However, we found significant somatic diversity within the FS substrain (Figure 3A), suggesting that the soma is not rapidly taken over by a single neoblast lineage in this substrain. Another possibility is that there is a hierarchical organization of stem cell potency, with only a fraction of neoblasts being truly pluripotent and thus capable of contributing to the genotype of the individual over long periods of time. However, Raz et al. have proposed a non-hierarchical model of neoblast potency, based in part on the ability of fate specified neoblasts to generate non-fate specified progeny.^70^ Thus, there is evidence to suggest that neither stem cell competition nor a potency hierarchy can fully explain the low heritability of somatic mutations in fissiparous planarians, leading us to propose that a genetic bottleneck driven by fission is likely to drive the low heritability of new mutations in the next generation via the tail.

### Generational mutation rates in asexual planarians

Diversity, both within and between individuals, depends on the rate of mutation, making it important to understand the rate at which mutations accumulate in the soma of asexual planarians. Planarian per generation mutation rates are driven by mutations in the neoblasts, which are the only cycling cells and lead to complete tissue turnover within two months.^38^^,^^39^ Previous research has shown that asexual *Dugesia japonica* planarians accumulate diversity rapidly, with nonsynonymous mutations being detected in 74% of genes after 20 years of clonal reproduction.^50^ Our results also support a high mutation rate in asexual *S. mediterranea*, with an estimated mutation rate of ∼3.75 × 10^−7^ per bp per generation. When considering only newly detected mutations that are also found in the next generation formed by the tail, the estimated mutation rate per generation is ∼3.31 × 10^−8^. Notably, we observed substantial variation in the number of newly detected mutations across individuals, which may be influenced by differences in offspring size and the time elapsed between fissions (Figure S11C). However, more samples will be needed to accurately estimate the impact of generation time on the per-generation mutation rate and to dissect whether other determinants such as offspring type (head vs. tail) or size also play a role.

While we provide estimates of per-generation mutation rates in asexual *S. mediterranea*, the absence of a single-cell bottleneck introduces an important caveat. *De novo* mutations arising in somatic cells may be present in only a small fraction of the organism’s total cell population, reducing our ability to detect them. As a result, our estimates may underrepresent the true mutation rate if many low-frequency somatic mutations go undetected, or overrepresent it if some mutations originated in earlier generations but were only recently detectable due to clonal expansion.

S*chmidtea polychroa*, which reproduces parthenogenetically through a single-cell bottleneck, has an estimated mutation rate of 1.31 × 10^−8^ per bp per generation.^71^ Interestingly, when *S. polychroa* individuals were amputated and allowed to regenerate before reproduction, the number of *de novo* mutations per generation increased nearly 3-fold. This link between regeneration and increased mutation rates may help explain the relatively high mutation rate observed in asexual *S. mediterranea* and may allow fissiparous species to generate additional genetic diversity in the absence of sex.

In summary, we showed that a population of clonal planarians previously thought to be homogeneous is composed of two distinct substrains which differ in genotype and phenotype. One substrain was significantly less fit than the other and contained more long runs of homozygosity. Additionally, we found that the less fit substrain had an unbalanced haplotype indicative of high levels of somatic diversity, which we suggest may be related to its reduced fitness. Finally, we find that fission likely acts as a genetic bottleneck, which may limit the accumulation of somatic diversity over time. The discovery of closely related planarian substrains that exhibit different somatic diversity and fitness creates an opportunity to understand the genetic basis of these traits in an obligately fissiparous animal and suggests that fissiparous populations can generate more diversity than is often assumed.

### Limitations of the study

One limitation of this study is the ∼50 cell rescue experiment. We wanted to generate a single-cell bottleneck via a single neoblast rescue as described in the study by Wagner et al.,^38^ but despite a few hundred attempts were not successful. To the best of our knowledge, there have only been two studies describing single neoblast rescue experiments, with low success rates and persistent health issues following rescue.^38^^,^^52^ One of these studies^38^ followed the reproductive behavior of the first few descendants of the rescued planarians. Here, we described the long-term rescue of lethally irradiated worms via bulk injections containing approximately 50 cells. While likely not all injected cells were totipotent neoblasts that would contribute to the rescue, the lack of a true single-cell bottleneck may be why we could not detect genetic differences between the non-perturbed and bottleneck lineages. Moreover, it was impossible to determine trends in genetic differences over time in the bottleneck lineages, as the early individuals within a lineage were needed to continue propagating the line. Mapping of these asexual *S. mediterranea* samples was performed against the sexual *S. mediterranea* genome,^72^ as this was the best assembly available at the time. Although there are known genomic differences between the two strains, it has been shown that the sexual genome can be appropriately used for asexual samples.^72^ Because all samples were mapped to the same genome, it is unlikely that our results are driven by any quality issues with the genome or assembly errors as these would be expected to equally affect all samples. Another concern is that calculation of the mutation rate was performed in individuals from the FS_B_ families, which we demonstrated have high somatic diversity. Thus, newly detected mutations could have been present in previous generations but at frequencies below our detection limit. Finally, it would have been interesting to study the accumulation of mutations for longer generations or periods of time but the intrinsic high death rate and slow reproduction rate of asexual planarians, especially of consecutive tail generations, made this unfeasible.

## Resource availability

### Lead contact

Requests for further information and resources should be directed to and will be fulfilled by the lead contact, Eva-Maria S. Collins (ecollin3@swarthmore.edu).

### Materials availability

This study did not generate new unique reagents.

### Data and code availability

• Data. The raw sequencing reads have been deposited on the NCBI Sequence Read Archive (BioProject: PRJNA1193082,^73^) and are publicly available as of the date of publication. Accession numbers are listed in the key resources table. All reproductive behavior data can be found in Dataset S1. All data reported in this paper will be shared by the lead contact upon request.
• Code. All original code has been deposited at Zenodo and is publicly available as of the date of publication. DOI is listed in the key resources table.
• Other Items. Any additional information required to reanalyze the data reported in this paper is available from the lead contact upon request.

## Acknowledgments

This work was funded by 10.13039/100000001National Science Foundation CAREER grant 1555109 (to E.-M.S.C.). Z.S. and V.S. were supported by a 10.13039/100012616Swarthmore College summer fellowship. The funders had no role in the design and conduct of the study, in the collection, analysis, and interpretation of the data, and in the preparation, review, or approval of the manuscript. The authors thank N. Campillo and J. Gayles for help with planarian maintenance, Drs. C. Lengner and L. Ni for use of their Gammacell 40 Exactor irradiator, Dr. D. Carone for use of her microscope, J. Paige for help with analysis of the population dynamics; Drs. D. Wagner, E. Siggia, B. Pearson, L. Guo, S. Duttke, E. Miller, and J. Carter for discussions; Dr. A. Griffin for discussions and comments, and Drs. R. Somach and S. Hackler for comments on the manuscript.

## Author contributions

Conceptualization and methodology, S.K., D.I., A.A., and E.-M.S.C.; investigation and formal analysis, S.K., D.I., V.S., Z.S., C.R., and E.-M.S.C.; writing – original draft, S.K., D.I., and E.-M.S.C.; writing – review and editing, S.K., D.I., V.S., Z.S., C.R., A.A., and E.-M.S.C.; funding acquisition, E.-M.S.C.; resources, E.-M.S.C.; project administration, E.-M.S.C.; supervision, A.A. and E.-M.S.C. All authors read and approved the final version of the manuscript.

## Declaration of interests

The authors declare no competing interests.

## STAR★Methods

### Key resources table

**Table:** 

REAGENT or RESOURCE	SOURCE	IDENTIFIER
**Chemicals, peptides, and recombinant proteins**		

Gentamycin	Omega Scientific	GT-03
Sigmacote	Sigma-Aldrich	SL2-25ML
Molecular Probes DAPI (4′,6 Diamidino 2 Phenylindole, Dihydrochloride)	Thermo Scientific	D1306

**Critical commercial assays**		

Wizard® Genomic DNA Purification Kit	Promega	A1120
Qubit™ dsDNA BR Quantification Assay Kit	Thermo Fisher Scientific	Q32850
KAPA HyperPrep Kit	Roche	KK8502

**Deposited data**		

Code	This study, zenodo.org	Zenodo: https://doi.org/10.5281/zenodo.14867003
Raw whole genome sequencing reads of individual planarians from different lineages	This study; NCBI Sequence Read Archive	BioProject: PRJNA1193082

**Experimental models: Organisms/strains**		

*Schmidtea mediterranea:* CIW4 asexual strain	E-M Collins laboratory	N/A
*Schmidtea mediterranea:* S2F2 sexual strain	E-M Collins laboratory	N/A

**Software and algorithms**		

Python	https://www.python.org/	version 3.9.5 RRID:SCR_008394
R	https://www.r-project.org/	versions 4.2.0 and 4.3.0 RRID:SCR_001905
SAPling	Thomas and Schötz^44^	N/A
Galaxy	https://usegalaxy.org/	RRID:SCR_006281
Plink	https://www.nitrc.org/projects/plink	RRID:SCR_001757
VCFtoolds	https://vcftools.sourceforge.net/	v0.1.16; RRID:SCR_001235
dNdScv	https://github.com/im3sanger/dndscv	RRID:SCR_023123
Bedtools	https://bedtools.readthedocs.io/en/latest/	RRID:SCR_006646
RStudio	https://posit.co/download/rstudio-desktop/	RRID:SCR_000432

**Other**		

Low-dose irradiator	Best Theratronics	Gammacell® 40 Exactor
40 μm cell strainer	Fisher Scientific	08-771-1
Compound microscope with 10× objective	Nikon	ECLIPSE C*i*
Borosilicate capillary tube (1.0 mm OD, 0.58 mm ID)	World Precision Instruments	1B100-4
Micropipette puller	Sutter Instrument	P-1000
Microinjection rig	World Precision Instruments	PV 820 Pneumatic PicoPump
Epifluorescent microscope with 100× oil objective	ZEISS	Axio Observer Z1 with Axiocam 702 mono camera

### Experimental model and subject details

#### Bulk maintenance of planarians

Sexual (S2F2) or asexual (CIW4) *Schmidtea mediterranea* planarians from established lab cultures were used for all experiments. As invertebrates, no institutional permission nor oversight for these animals was required. Planarians of various “ages” (time since last division) were used as this was not a controlled variable.

According to established protocols,^43^ planarians were maintained in 1× Montjuic Salts.^74^ When not used in experiments, the planarians were stored in a Panasonic refrigerated incubator at 20°C in the dark. The planarians were fed 1–2X per week with grass-fed beef liver (purchased from a local farm) or certified organic chicken liver (Bell and Evans). The planarians’ containers were cleaned on the day of feeding, 1–2 h after feeding, and 2 days later. Planarians were not fed for at least 5 days prior to being used in experiments.

#### Generation of bottleneck planarians

The bottleneck planarians were generated following published methods,^38^^,^^51^ with a few modifications. To generate the bottleneck asexual *S. mediterranea*, sexual *S. mediterranea* planarians were lethally irradiated with 93 Grays (9,300 rads) on a Gammacell 40 Exactor irradiator (Best Theratronics, Springfield, VA). After irradiation, sexual planarians were kept in 10 cm plastic petri dishes (Simport Scientific) in 1× Montjuic Salts supplemented with 50 μg/mL gentamycin (Omega Scientific, Tarzana, CA, USA). Two days following irradiation, irradiated hosts were injected with ∼50 cells/planarian from an asexual *S. mediterranea* donor. The donor had been fasted for at least 7 days before dissociation into individual cells. The donor planarian was placed in a 1.7 mL microcentrifuge tube with 1 mL Calcium/Magnesium-Free Medium (“CMF”) + 1% BSA (CMFB)^51^ and by pipetting up and down with a P1000 for 5 min. The dissociated cells were filtered with a 40 μm cell strainer (Fisher Scientific, Hampton, NH) and subsequently centrifuged at 290 x*g* for 5 min at 4°C. The pellet was resuspended in 0.5 mL CMFB. Roughly 20 μL of the cell suspension was spotted onto an uncoated glass coverslip and inspected using a Nikon Eclipse C*i* compound microscope at 10× magnification. While avoiding cell clumps, individual cells from a region of high density on the coverslip were mouth pipetted using a borosilicate glass needle. The needle was prepared from a capillary tube (World Precision Instruments, product #1B100-4 1.0 mm OD, 0.58 mm ID) using a Sutter Instrument P-1000 micropipette puller and was pretreated with Sigmacote for 5 min before use. The number of cells injected (∼50) was determined manually by counting the number of cells entering the needle during the aspiration, using the microscope. The cells were injected by sticking the needle through the tail stripe on the ventral side of the sexual hosts and pointing the needle toward the parenchymal space using 1.5–2.5 psi pressure on an injection rig using a WPI PV 820 Pneumatic PicoPump. Following injection, the injected planarians were kept individually in 10 cm petri dishes containing 50 μg/mL gentamycin in 1× Montjuic Salts and cleaned every 2–3 days in the dark to avoid light exposure which can lead to reduced life spans.^51^

Two out of 180 irradiated sexual planarians that were injected with asexual neoblasts survived. These two rescued planarians (B1 and B2) began showing signs of regeneration approximately 43 days after the rescue and divided 82 days after the rescue. They were confirmed to have been converted to asexual using karyotype analysis (Figure S6A). Tail snips from the injected planarians were used for chromosome preparation as described in.^75^ Slides with chromosome spreads were stained with DAPI (Molecular Probes, Thermo Fisher Scientific, Waltham, MA, USA) and imaged using a ZEISS Axio Observer Z1 epifluorescent microscope equipped with a ZEISS Axiocam 702 mono camera with a ×100 oil objective. Ten chromosome spreads from each tail snip were analyzed for the presence of the asexual chromosome translocation. Tail snips from one asexual and one sexual *S. mediterranea* were used as controls.

#### Planarian individual maintenance

The progeny of the two rescued individuals were maintained and tracked. Individual planarians were maintained in 10 cm petri dishes (Simport Scientific, QC, CA) in approximately 25 mL 1× Montjuic Salts per worm in the same incubator in the dark, according to the procedures described in.^37^^,^^41^^,^^42^^,^^43^^,^^44^ In brief, individual planarians were fed and cleaned following the same schedule as bulk planarians and were checked every 2–3 days for divisions. Divisions were recorded in the SAPling database^44^ and offspring separated into new dishes. Each planarian container had a unique QR-code identifier.^44^ Certain individuals (Table S1) were flash frozen and used for whole genome sequencing. The bottleneck lineages were maintained in his way for 2.5 years (31 months) until the termination of this experiment.

### Method details

#### Comparison of asexual planarians from different “lineages”

We utilized existing data from previous experiments^37^^,^^41^^,^^42^^,^^43^^,^^44^ tracking the reproductive dynamics of asexual *S. mediterranea* “lineages”. Each lineage originated from a single planarian founder arbitrarily chosen from an established lab culture of asexual *S. mediterranea* planarians (CIW4 strain) (Figure S1A). The planarians were allowed to naturally reproduce asexually and all subsequent offspring were cultured in individual petri dishes (see “planarian individual maintenance”) and their behavior (reproduction, death) was tracked with a custom barcode system.^44^ We used data from four of these lineages: Fritz (F), Sophie (S), Peter (P) and Hans (H) (Dataset S1).

To compare the genetic identity of these closely related individuals, we utilized previously frozen individual planarians for genomic sequencing. To allow for direct comparisons between lineages and minimize potential bias due to culturing length or worm identity, corresponding individuals from the same generational history were used for sequencing (Figure S1B and Table S1). For each generational timepoint, samples were compared from at least two different non-bottleneck lineages and from the two bottleneck lineages. Thus, 4–5 samples from each non-bottleneck lineages and 8 samples from each bottleneck lineage were sequenced. In some instances, the desired individual was created by freezing its two offspring within 2 days of division.

### Next generation whole genome sequencing

Individual planarians were flash frozen in liquid nitrogen and stored at −80°C prior to genomic DNA extraction using a Wizard Genomic DNA Purification Kit (Promega, Madison, WI). The protocol for Isolation of Genomic DNA from Animal Tissue and Tissue Culture Cells was used, including sample lysis for 30 min at 55°C with shaking. Genomic DNA yield was measured using a Qubit dsDNA BR assay (Thermo Fisher Scientific, Waltham, MA).

Library preparation and next generation whole genome sequencing was performed by Admera Health using a KAPA HyperPrep Kit (Roche, Indianapolis, IN) and Illumina NovaSeq sequencer (paired 150 bp reads, ∼200 M paired end reads per sample), respectively. The raw sequencing reads have been deposited on the NCBI Sequence Read Archive (BioProject: PRJNA1193082:^73^).

### Sequencing pre-processing and variant calling

Sequencing data was uploaded to the Galaxy web platform and the public server usegalaxy.org was used to create the trimmed and mapped reads.^76^ The quality of the reads was checked using fastp,^77^ which also performed adaptor trimming. Reads were mapped to the reference sexual *S. mediterranea* genome^72^ using BWA-MEM.^78^ The quality of the mapped reads was checked with ValidateSamFile and duplicates were marked using MarkDuplicates. Variant detection was performed using GATK^79^ using the gVCF setting across all gene bodies (annotation file from^80^). Variants were quality filtered using GATK suggested parameter values.^81^ Only variants with a depth between 0.5 and 2 times the mean depth were included. All variants that were found in all individuals either as heterozygous or homozygous were removed by filtering out those with an allele frequency >0.5. All variants found within repetitive regions (defined in a previous publication^23^) were removed from the analysis.

To cluster the different populations, Plink^82^ was used to prune the data and run Principal Component Analysis. Pruning was done with a sliding window of 50 SNPs, a step size of 10 SNPs, and an r^2^ threshold of 0.1. Outliers were identified in the PCA and upon inspection were removed from the analysis since they contained high levels of uncovered variants (Figure S12). To minimize the impact of depth variation on PCA clustering, we restricted the analysis to sites genotyped with over 99% confidence (GQ > 20) across all included samples.

### FST and nucleotide diversity

We used VCFtools (v0.1.16) to calculate the mean pairwise fixation index scores (FST) between each pair of lineages and substrain, with non-overlapping window sizes of 10,000 bps. Pairwise FST scores between lineages were calculated using only sites genotyped with high confidence (GQ > 20) across all analyzed samples. To calculate differences in nucleotide diversity (mean number of nucleotide differences per site) between the PH and FS substrains, we ran a nucleotide diversity test within each substrain using VCFtools with non-overlapping window sizes of 10,000 bps. To understand whether we see reduced diversity in the populations derived from bottleneck worms, we also ran a pairwise nucleotide diversity test between all libraries within each lineage using similar settings. We then asked whether bottleneck information is a good predictor of nucleotide diversity when also including information about mean sample depth (and the interaction between these two variables) (Figure S2).

### Runs of homozygosity

Runs of homozygosity were assessed using Plink, searching for regions that are at least 50 Kb long, containing at least 25 SNPs, with neighboring SNPs being at most 50 Kb from one another. Bedtools merge and intersect were used to identify FS- and PH-specific runs of homozygosity.^83^ First, runs of homozygosity in different individuals within each lineage were merged to form a single set of coordinates for the run of homozygosity. Then, if all individuals within a lineage supported the merged run of homozygosity, it was annotated as a run of homozygosity found within the lineage. Finally, bedtools intersect was used to isolate runs of homozygosity that were specific to either the PH or FS substrains. A list of the runs of homozygosity in each sample is listed in Dataset S2. Bedtools nuc was used to calculate the GC content within and outside runs of homozygosity.^83^ To assess whether the FS-specific runs of homozygosity are found in other laboratories across the world, we used previously published data about the variants present in RNAseq libraries from different BioProjects.^23^ Each variant in this dataset has an associated allele frequency within its BioProject, so we plotted the mean allele frequency of each variant in our region of interest to see if there is a deviation from the expected allele frequencies (1 or 0.5), which would indicate mixed zygosity in the population. Although all reads were initially mapped to a scaffold-level genome,^71^ we used a more recent chromosome-level assembly to identify the chromosomal locations of runs of homozygosity, utilizing a scaffold-to-chromosome mapping file provided by Guo et al.^65^ Ambiguously mapped scaffolds were excluded from this analysis.

#### dNdScv

To assess the impact of variants on protein sequence and isolate genes and isolate genes containing non-synonymous/frameshift mutations between the FS and PH substrains we ran dNdScv.^84^ The dNdScv annotations for all FS and PH SNPs and indels are listed in Datasets S4 and S5. We used Orthofinder^83^ to annotate the potential human and *Drosophila melanogaster* orthologues of genes containing non-synonymous and nonsense variants (Dataset S6).

#### Somatic allele frequency

To assess the frequency of detected mutations in the soma, we divided the allele depth (AD) supporting the alternative variant by the total allele depth at the site (Depth_Alternative_ / (Depth_Alternative_ + Depth_Reference_)). A heterozygous variant that is found in all cells should have an allele frequency of 0.5. Only variants with a total support of at least 10 reads were used for such calculations (sum of allele depths >9).

#### Generation of “head lines” and calculation of mutation rate

For a subset of the progeny from the two bottleneck planarians, for consecutive divisions we retained the tail offspring and froze the head offspring (Table S2). Note that *S. mediterranea* tail offspring rarely fragment.^37^ All divisions consisted of fission events. We chose to retain the tails because they are the smaller offspring (Figure S9A) and must regenerate more complicated structures, including the brain, thus requiring more stem cell divisions. However, tails have a much higher death rate (Figures 1E and S8C) and longer reproductive waiting times than heads (Figure S9B); thus, we were only able to collect 3–4 consecutive head offspring for each tail line, resulting in 5 separate lineages for a total of 16 samples. Of these 5 lineages, one was discarded since it contained an outlier sample with high levels of uncovered variants (Figures S12C and S12D). For some of the lines, intermediate heads divided before they could be frozen and thus not all head lineages were complete consecutive lines.

To quantify the number of newly detected mutations that arose in each generation, we identified all SNPs that were present in a daughter worm, but not in its parent. We then excluded all SNPs that were found in other libraries, excluding any of the progeny of the daughter worm (since a *de novo* mutation may be passed on to the next generation), and SNPs that were found within a single read length (150 bps) from other putatively *de novo* mutations. We also removed any variants that were found in other datasets, including the non-perturbed dataset and those found in other publications.^23^ Additionally, we only considered newly detected mutations whose position was covered by all libraries to make sure that they were truly absent from other libraries. These filtering steps make it highly unlikely that newly detected mutations that are passed on to the next generation are due to recurrent sequencing errors. By plotting the cumulative number of discarded putative *de novo* mutations per cross referenced library, we show that after cross referencing, most remaining putative *de novo* mutations are likely to be truly unique mutations (Figure S10). We compared the somatic allele frequency of *de novo* mutations to established mutations (see above). In this comparison, we defined established background mutations as those found in both PH and FS substrains. To calculate the mutation rate per generation per bp, we divided the number of newly detected mutations by the number of bps examined (148,090,923 non-repetitive loci within gene bodies), multiplied by two due to the diploid nature of asexual *S. mediterranea* resulting in 296,181,846 potential sites for detecting mutations. Since our analysis was limited to variants within non-repetitive gene body regions, the number of base pairs examined reflects only those within non-repetitive segments of gene bodies. Repetitive regions were removed from a BED file of gene body coordinates using bedtools subtract and overlapping intervals in the resulting file were consolidated with bedtools merge to produce a final BED file representing all non-redundant, non-repetitive base pairs within gene bodies.

### Quantification and statistical analysis

For all statistical analysis, *p*-values <0.05 were considered statistically significant. Details on the statistics performed for specific analyses can be found in the main text and respective figures.

#### Population growth dynamics

The reproductive dynamics of the F, S, H and P lineages (“non-perturbed lineages”) were obtained from previous experiments.^37^^,^^41^^,^^42^^,^^43^^,^^44^ Data from the non-perturbed lineages were truncated to only the first 31 months to match the duration the bottleneck lineages were cultured to allow for fair comparisons. Original data organization and calculation of reproductive waiting times was performed in Python. Quantitative and statistical analysis was performed in R (version 4.3.0,^85^). Reproductive waiting times were calculated as the time from the birth of an individual to its subsequent division using the data from the SAPling system.^37^^,^^41^^,^^42^^,^^43^^,^^44^ Statistical analysis of the reproductive waiting times of all division events were performed using a Kruskal-Wallis omnibus test followed by Dunn’s post hoc test with *p*-value adjustment with the Bonferroni method using the rstatix package.^86^ Fragmentations were defined as divisions that occurred with a reproductive waiting time <6 days.

Multi-offspring fragmentations, i.e., consecutive rounds of divisions each with reproductive waiting times <6 days, were aggregated together as one fragmentation event. Comparison of the relative frequencies of fission vs. fragmentation was performed using a multi-comparison Fisher’s exact test with *p*-value adjustment using the Bonferroni method from the RVAideMemoire R package.^87^ To statistically compare the distribution of the number of middles created during fragmentations, a generalized linear model using a Poisson distribution was created to test the influence of lineage on the number of middles using the stats package.^85^ As lineage was found to be a significant predictor of the number of middles, we performed pairwise comparisons of the estimated marginal means with *p*-value adjustment using the Bonferroni method using the emmeans package.^88^

The percentage of deaths in a lineage was calculated as the number of deaths out of the total numbers of planarians that either died or divided (since we only have data on individuals when they do either of these). Frequencies of death were calculated for heads and tails separately. Statistical analysis for each offspring type were performed using a multi-comparison Fisher’s exact test with *p*-value adjustment using the Bonferroni method from the RVAideMemoire R package.^87^

The raw population data can be found in Dataset S1.

#### Genetic analysis

To assess the relatedness among lineages, we compared pairwise FST scores between lineage pairs (Figure 2B) using a paired Wilcoxon test, matching mean FST values across the same scaffolds for each lineage pairing, with Bonferroni *p*-value adjustment to adjust for multiple testing. To compare the number of runs of homozygosity in the FS and PH substrains, we used a Wilcoxon test with each sample being used as a replicate (Figure 2E). We then used a paired Wilcoxon test to assess whether runs of homozygosity were GC-biased and whether there were differences in depth within runs of homozygosity compared to outside runs of homozygosity (all comparisons were paired by scaffold; Figures S3C–S3E). To compare the allele frequency of FS and PH specific variants within the soma, we used a Wilcoxon test using each SNP as a datapoint (Figure 3A). Using a Wilcoxon test with Bonferroni *p*-value adjustment to account for multiple testing, we also assessed whether differences in contamination levels differ between the FS and PH substrains (Figure S6B). A Wilcoxon test was also performed to assess whether FS-specific mutations had higher depth than FS mutations shared with PH (Figure S6C). To test whether FS-specific mutations that were found in all FS individuals also showed allelic imbalance, we also ran a Wilcoxon test (Figure 3C). To test whether the individuals derived from the bottleneck lineages have reduced nucleotide diversity we ran a quasi-binomial generalized linear model, with the predictors including mean sample depth, bottleneck lineage (TRUE/FALSE), and the interaction between the terms (Figure S2C). Additionally, a Wilcoxon test with a Bonferroni adjustment was used to compare the heterozygosity levels of all lineages. Finally, when assessing whether putative *de novo* mutations differ in their allele frequency within the soma, we used a Wilcoxon test.

## Supplemental information

Document S1. Figures S1–S12 and Tables S1–S3

Dataset S1. Raw population data

Dataset S2. Runs of homozygosity per sample

Dataset S3. List of substrain-specific variants found in all individuals of each substrain

Dataset S4. dNdScv annotation output for “Family” dataset SNPs

Dataset S5. dNdScv annotation output for “Family” dataset INDELs

Dataset S6. List of susbtrain-specific non-synonymous and nonsense mutations annotated with Human (Hsa) and *Drosophila melanogaster* (Dmel) orthologues found using OrthoFinder
