# Supplementary material for: Variation in phenotype, genotype, and somatic diversity among asexual Schmidtea mediterranea planarians
Source: iScience. 2025 Jul 1;28(8):113035. doi: 10.1016/j.isci.2025.113035 (PMC12284295; doi:10.1016/j.isci.2025.113035)
Supplement: Document S1. Figures S1–S12 and Tables S1–S3 [file mmc1.pdf]

iScience, Volume *28*

Supplemental information

Variation in phenotype, genotype, and somatic diversity among asexual *Schmidtea mediterranea* planarians

Simon Kershenbaum, Danielle Ireland, Ziad Sabry, Christina Rabeler, Vir Shetty, Aziz Aboobaker, and Eva-Maria S. Collins

Supplemental Figures

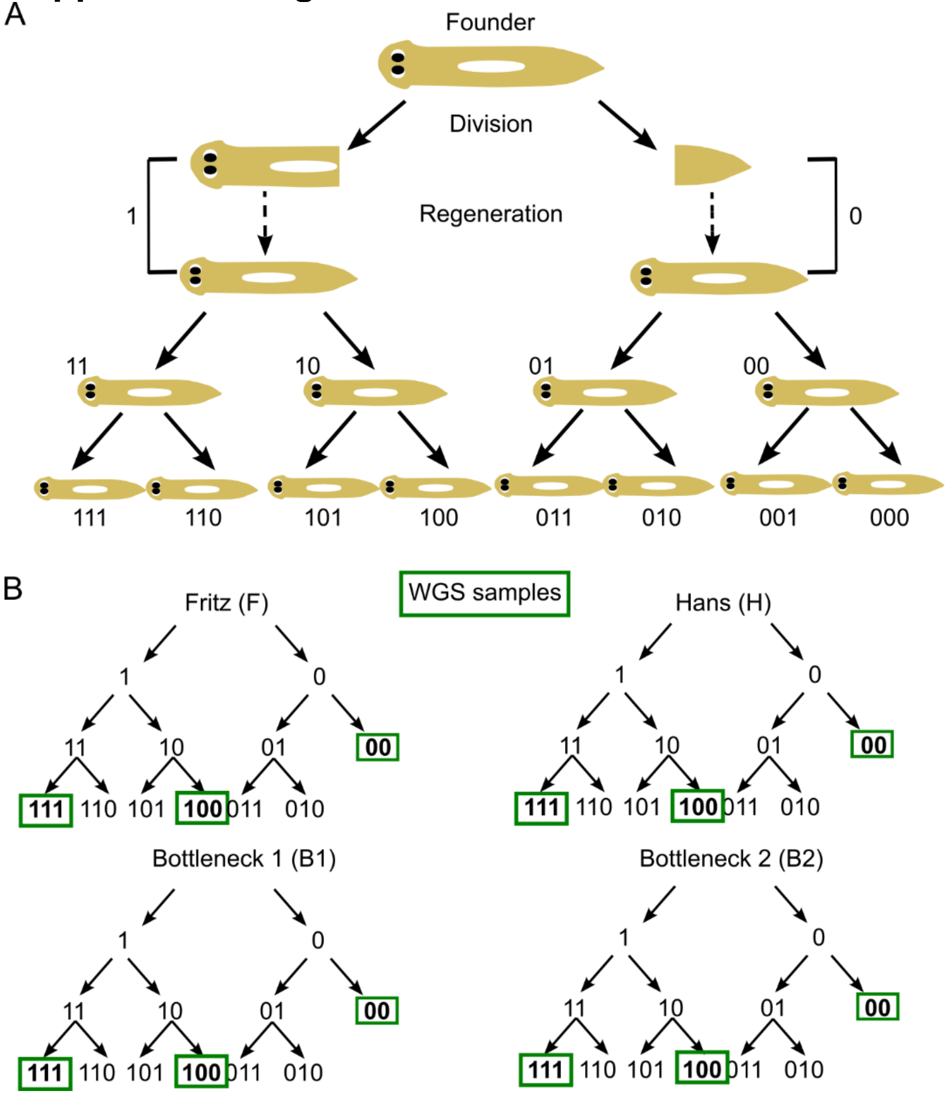

Figure S1. Schematic of creation of planarian lineages and sequencing scheme, related to Figure 1 and STAR Methods. A) Each planarian lineage was originally created from a single founder planarian arbitrarily selected from an established lab culture of asexual *S. mediterranea* (CIW4 strain). The founders were allowed to reproduce asexually, followed by subsequent regeneration into a full adult planarian (dashed arrow). This was repeated over many generations (each row) with all offspring cultured in individual petri dishes. The identity of each worm was tracked using a custom barcode system [S1] wherein head offspring are labelled as '1' and tail offspring are labelled as '0'. Middle pieces arise when the head offspring divides at least one more time after generation of the first tail in ≤ 5 days, before the head has fully regenerated (see Figure 1A). Trunk pieces are also denoted as "0" and can be distinguished from true tails through the time between divisions. For a detailed description of the lineage generation, see references [S1–S5]. The generational history of a worm consists of the identities of each division from oldest (left) to newest. Only the first three generations are shown for illustrative purposes, but the lineages were allowed to propagate for several years. Only the first 2.5 years of data are used in this study to keep comparisons similar to the Bottleneck lineages. B) From the lineages described in A, a subset of planarians had been previously frozen that could be used for whole genome sequencing (WGS). Because freezing a planarian terminates that branch of the tree and precludes the ability to get more population data (see worm 00), only a few individuals were frozen for sequencing. To remove potential bias due to generational history or duration of culturing, we compared worms from different

lineages at the same generational history (green boxes). For a given generational history, worms were chosen from two non-bottleneck lineages and the two bottleneck lineages (green boxes). The figure shows hypothetical examples to illustrate this idea. See Table S1 for a list of sequenced samples.

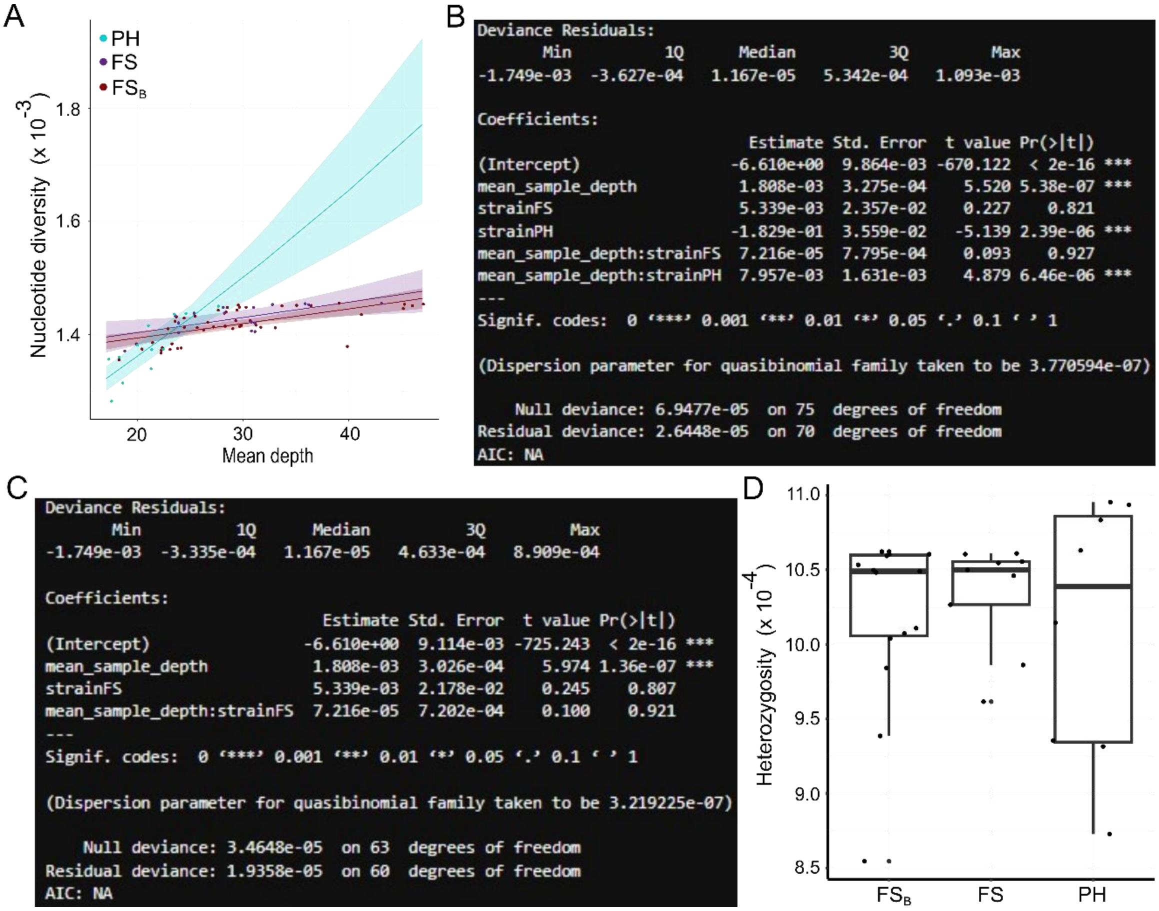

Figure S2. Comparison of nucleotide diversity and mean depth, related to Figures 1 and 3. A) Plot of mean nucleotide diversity per scaffold as a function of sample depth in all lineages. B) A quasibinomial generalized linear model found a significant interaction of mean depth and nucleotide diversity in the PH lineages, given its lower overall depth compared to the other lineages. Because of this we could not directly compare the nucleotide diversity of PH to the other lineages. Shaded regions represent 95% confidence intervals. C) Full statistical results of quasibinomial generalized linear model where only comparing FS and FS_B as shown in Figure 3D. D) There is no differences in overall heterozygosity levels between the lineages, with all groups having heterozygosity levels of 0.001 per site (p=1; Wilcoxon rank sum with Bonferroni p-value adjustment).

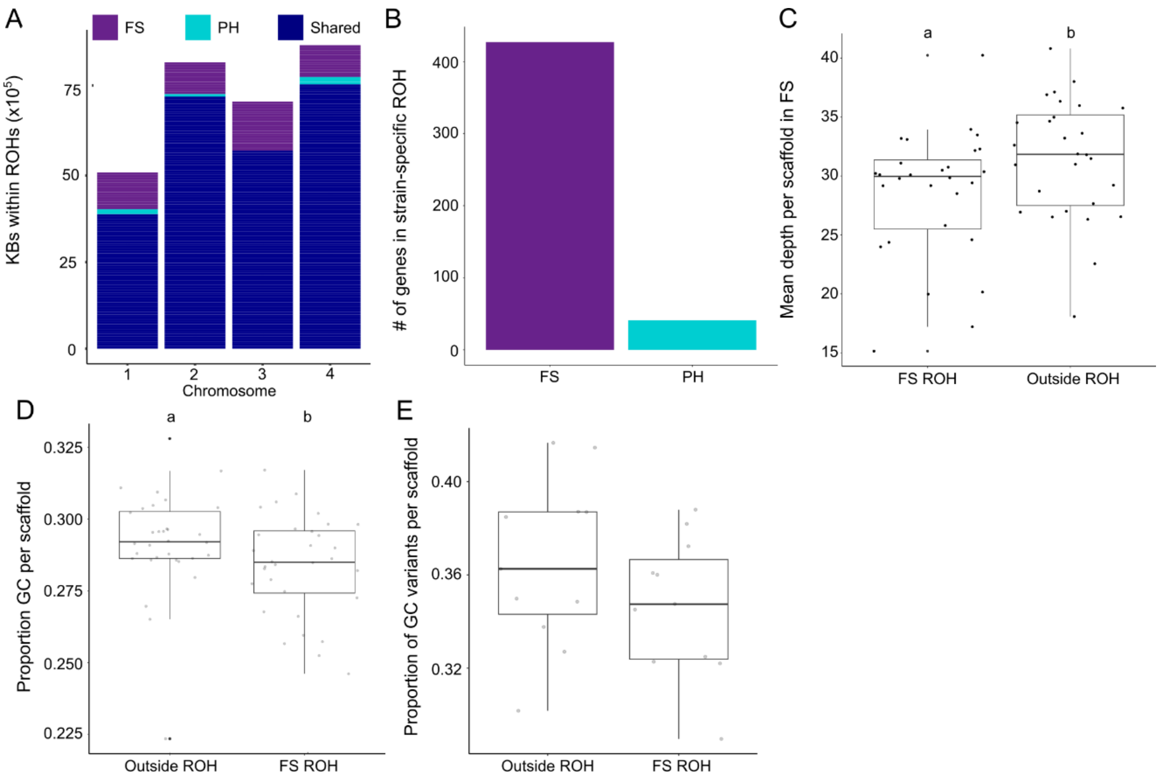

Figure S3. Deletions and gene conversion cannot fully explain the FS-specific runs of homozygosity, related to Figure 2. A) Runs of homozygosity (ROHs) were found on all chromosomes. Analysis was done as described in the STAR Methods using the sexual *S. mediterranea* genome published by Guo et al. [S6]. Runs of homozygosity are color-coded by whether they were found in both substrains (shared) or specific to either the FS or PH substrain. B) The FS substrain contains more genes in substrain-specific runs of homozygosity than PH. C) Mean depth per scaffold in FS-specific runs of homozygosity is significantly lower than outside of runs of homozygosity but is not low enough for deletions to be the driving force for the runs of homozygosity. Different letters indicate statistically significant differences ($p < 0.01$; Wilcoxon signed rank; $V = 99$, $N = 30$, $Mean_{FS\text{-}ROH} = 28.6$, $Mean_{Outside\text{-}ROH} = 31.6$). D) FS-specific runs of homozygosity are AT -rich rather than GC-rich, suggesting that GC-biased conversion is not the main contributor to these FS-specific runs of homozygosity ($p < 0.01$; Wilcoxon signed rank; $V = 127$, $N = 32$, $Mean_{FS\text{-}ROH} = 0.28$, $Mean_{Outside\text{-}ROH} = 0.29$). Each datapoint is the mean value per scaffold. E) Variants within FS-specific runs of homozygosity are not GC-biased, further suggesting that gene conversion is not the main contributor to these runs of homozygosity ($p = 0.102$; Wilcoxon signed rank; $V = 14$, $N = 11$, $Mean_{FS\text{-}ROH} = 0.35$, $Mean_{Outside\text{-}ROH} = 0.37$).

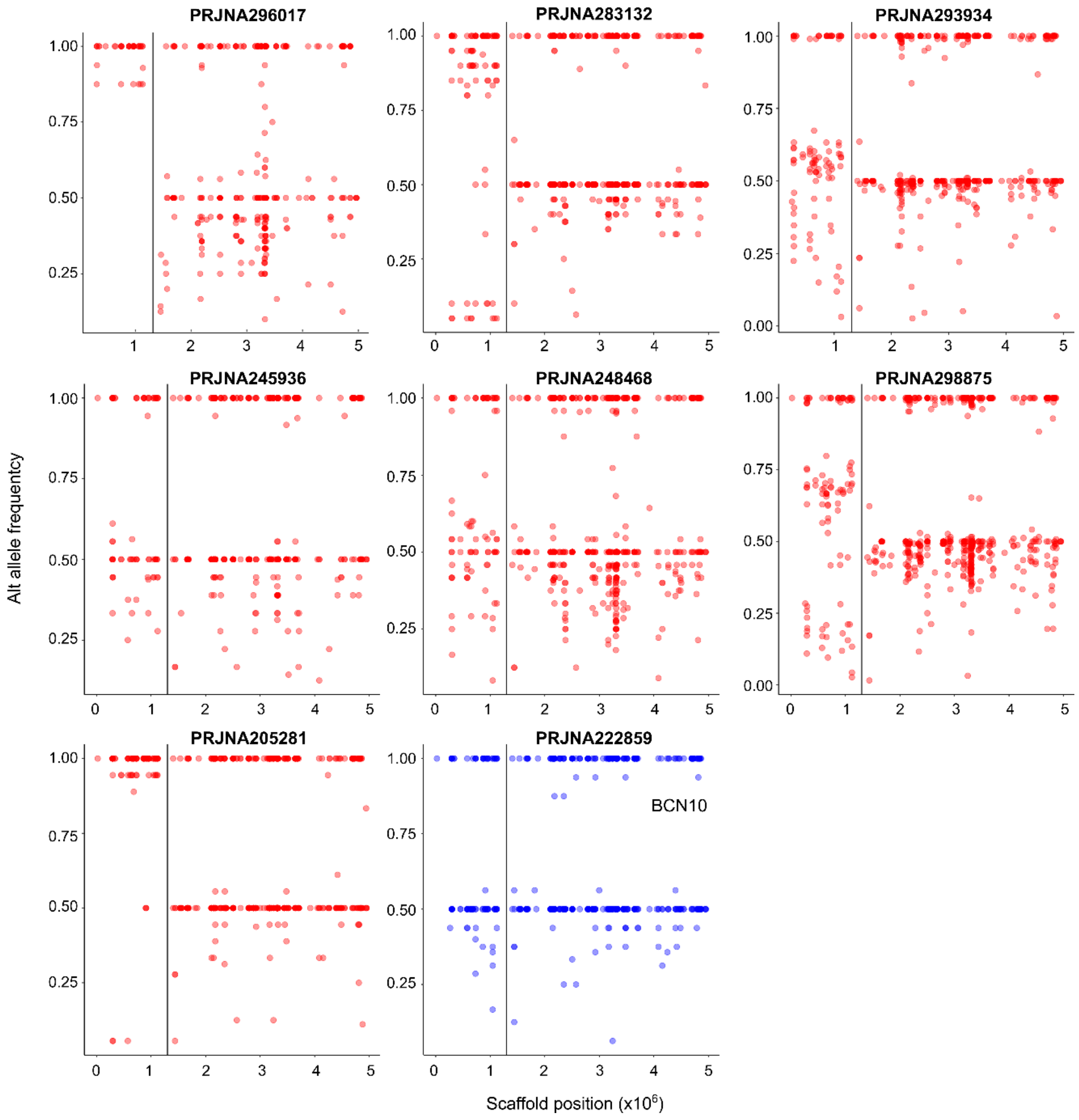

Figure S4. CIW4 planarians from other laboratories contain both FS and PH substrains, related to Figure 2G. A mixture of FS and PH substrains are found in various, but not all, CIW4 populations. Allele frequency of variants in the dd_Smes_g4_1 scaffold from different BioProjects (stated above each plot). The plot with blue points is from a BCN10 strain, which seems to lack individuals with an FS-like genotype. The dashed line indicates the end of the run of homozygosity.

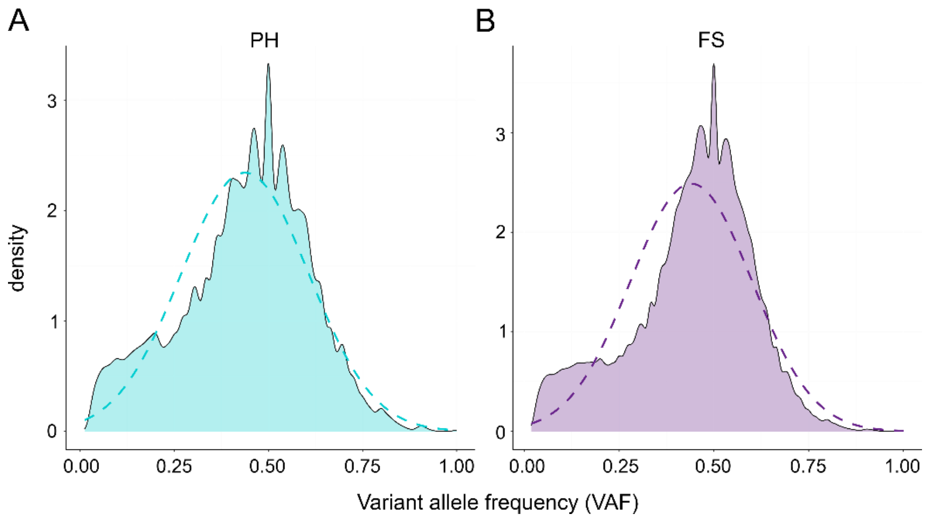

Figure S5. Distribution of variant allele frequencies in PH and FS, related to Figure 3. A) PH and B) FS show similar distributions of allele frequencies when comparing all variants found within each group. There is a clear peak at 0.5, representing variants that are heterozygous within all the cells in the body of individual planarians. This suggests that the soma does not consist of distantly related clones. However, there is also an excess of SNPs in both substrains that show low variant allele frequencies, which are likely to contain variants that are found only in a subset of cells (See Figure 3A for further analysis of these). The dashed line represents a normal distribution fitted to the data, showing that it is slightly left-shifted ($mean_{PH}$ = 0.438, $mean_{FS}$ = 0.440).

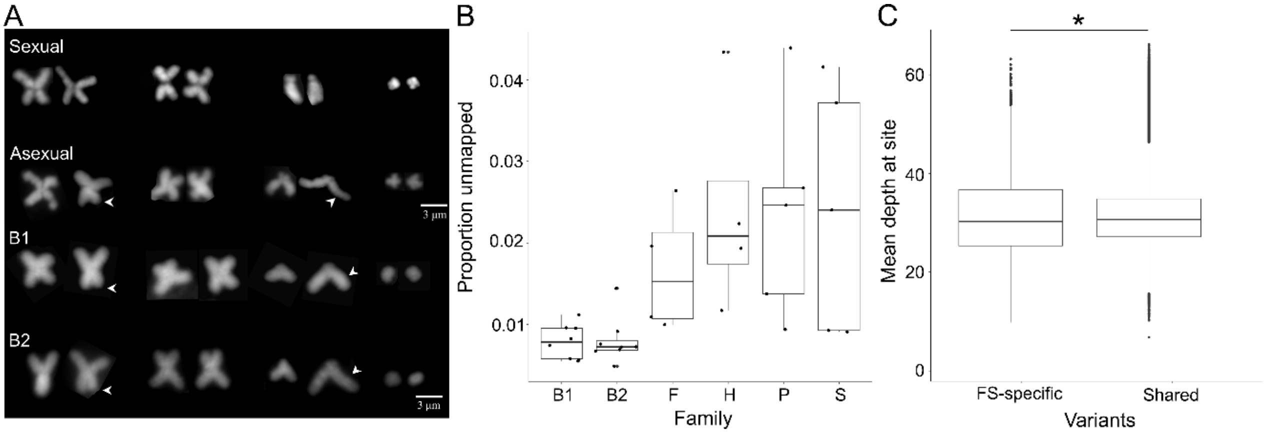

Figure S6. Allelic imbalance in FS-specific variants is not due to polyploidy, local duplications, or contamination, related to Figure 3). A) Karyotyping on the bottleneck lineages (which are members of the FS substrain, see Figure S7) shows a normal diploid karyotype. The bottleneck lineages show the asexual chromosomal translocation (arrow heads), demonstrating successful rescues by the asexual donor cells. Scale bar: 3 µm. B) Libraries have good mapping rates that are not indicative of a contamination problem. There are no significant differences across lineages ($p<0.05$, Wilcoxon rank sum with Bonferroni p-value adjustment; $N_{B1}=8$; $N_{B2}=8$; $N_F=4$; $N_H=4$; $N_P=5$; $N_S=5$). C) FS-specific variants have higher sequencing depth than shared variants in the FS substrain, suggesting that the allelic imbalance is not due to local duplications ($p<0.001$; Wilcoxon rank sum; N_{FS}-specific = 3684, N_{shared} = 170549, W = 303804886).

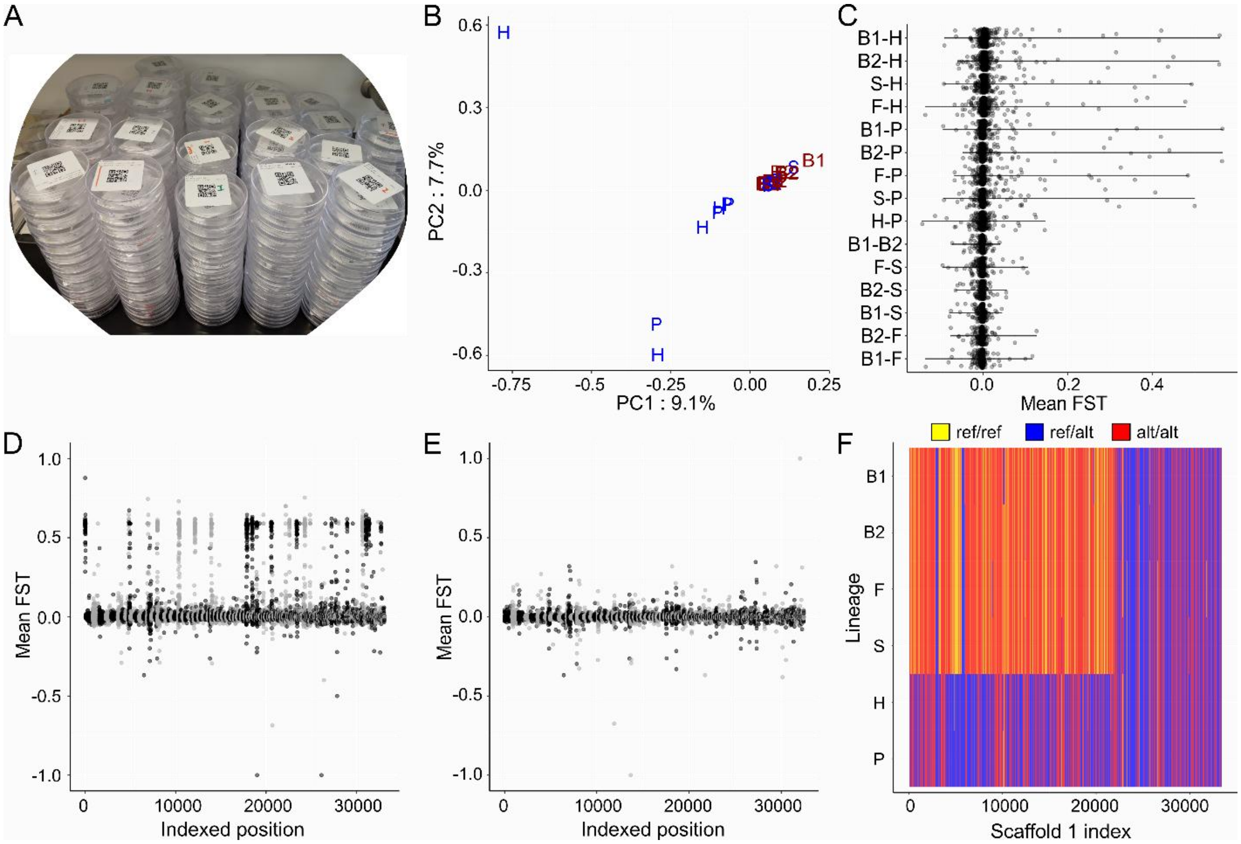

Figure S7. The bottleneck lineages come from the FS substrain, related to Figure 3. A) Picture of the individually cultured planarians generated from the two bottleneck founder planarians. The SAPling barcode system [S1] was used to track the reproductive events of each individual. B) PCA showing that the bottleneck lineages (in red) cluster with F and S. The letters indicate the lineages (P, H, F , S, B1, B2). Blue indicates the non-bottleneck lineages and red indicate the bottleneck lineages.C) Comparisons of FST scores between B1/B2 and FS are lower than compared to PH. D) FST scores between the bottleneck lineages and PH show similar peaks to those seen between FS and PH. E) There are no peaks of high genetic divergence between the bottleneck populations and FS. F) The bottleneck lineages contain the same run of homozygosity in scaffold dd_Smes_g4_1.

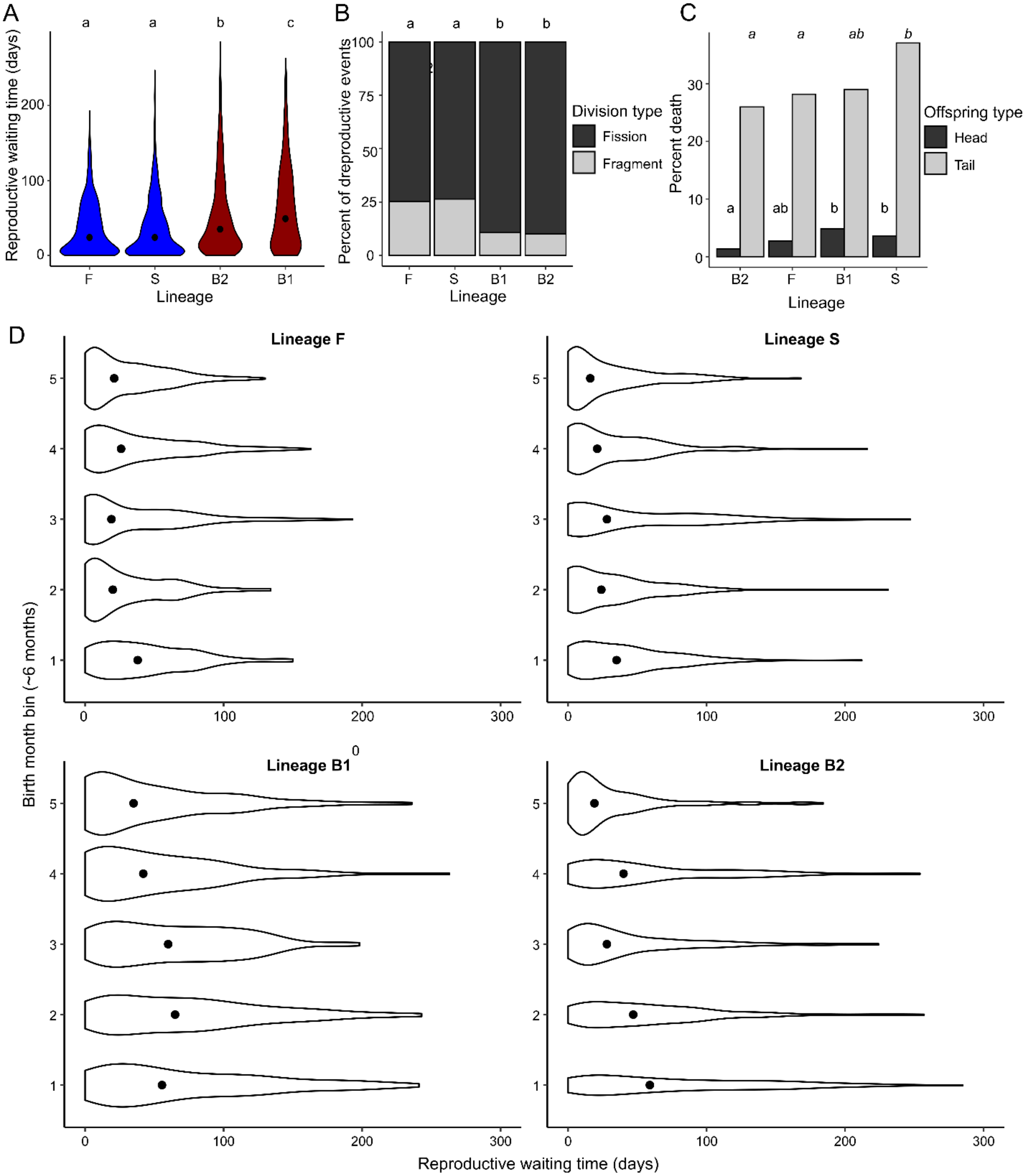

Figure S8. Reproductive dynamics of the bottleneck lineages, related to Figure 3. A) Violin plots of reproductive waiting time (time from birth to division) considering all divisions for each lineage. Lineages are in order of increasing median reproductive waiting time (dot). Different letters indicate statistically significant groups ($p<0.05$, Kruskal-Wallis omnibus test followed Dunn's posthoc test with Bonferroni p-value adjustment; $N_F = 1249$, $N_S = 953$, $N_{B2} = 953$, $N_{B1} = 718$). B) Percent of reproductive events that were fissions or fragmentations for each lineage. Different letters indicate statistically significant groups ($p<0.05$, Fisher's exact test with Bonferroni p-value adjustment; $N_F = 945$, $N_S = 701$, $N_{B2} = 859$, $N_{B1} = 639$). C) Percent death in each lineage for head

and tail offspring. Percent is out of the total number of worms that died or divided. Different letters indicate statistically significant groups with separate comparisons across head (non-italicized) or tail (italicized) offspring ($p<0.05$, Fisher's exact test with Bonferroni p-value adjustment; Heads: $N_{B2} = 663$, $N_F = 847$, $N_{B1} = 456$, $N_S = 705$; Tails: $N_{B2} = 404$, $N_F = 592$, $N_{B1} = 400$, $N_S = 434$). D) Reproductive waiting time varies with time for all lineages, when worms are binned based on their relative birth month from start of their respective lineage ($p<0.01$ for each family, Kruskal-Wallis test, $N_{F1} =188$, $N_{F2} =287$, $N_{F3} =261$, $N_{F4} =245$, $N_{F5} = 268$, $N_{S1} =171$, $N_{s2} =197$, $N_{s3} =163$, $N_{s4} =183$, $N_{F5} = 239$, $N_{B1-1} =124$, $N_{B1-2} =121$, $N_{B1-3} =141$, $N_{B1-4} =181$, $N_{B1-5} = 151$, $N_{B2-1} =169$, $N_{B2-2} =176$, $N_{B2-3} =221$, $N_{B2-4} =194$, $N_{B2-5} = 193$). The birth month data is split into 0.2 quantiles which represent approximately every 6 months (0.2 *31 months). Differences in reproductive waiting time distributions between the bottleneck and non-perturbed lineages become smaller over time.

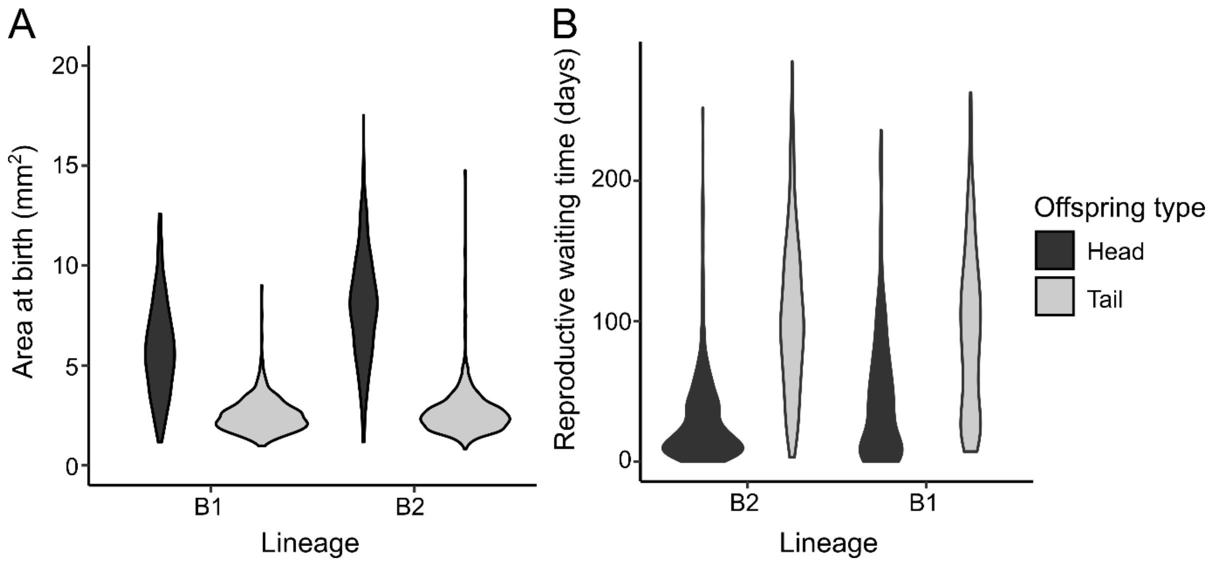

Figure S9. Comparison between head and tail offspring from bottleneck lineages, related to Figure 4 and STAR Methods). A) Tail offspring are born smaller than head offspring. B) Tail offspring have longer reproductive waiting times (time from birth to division) than head offspring.

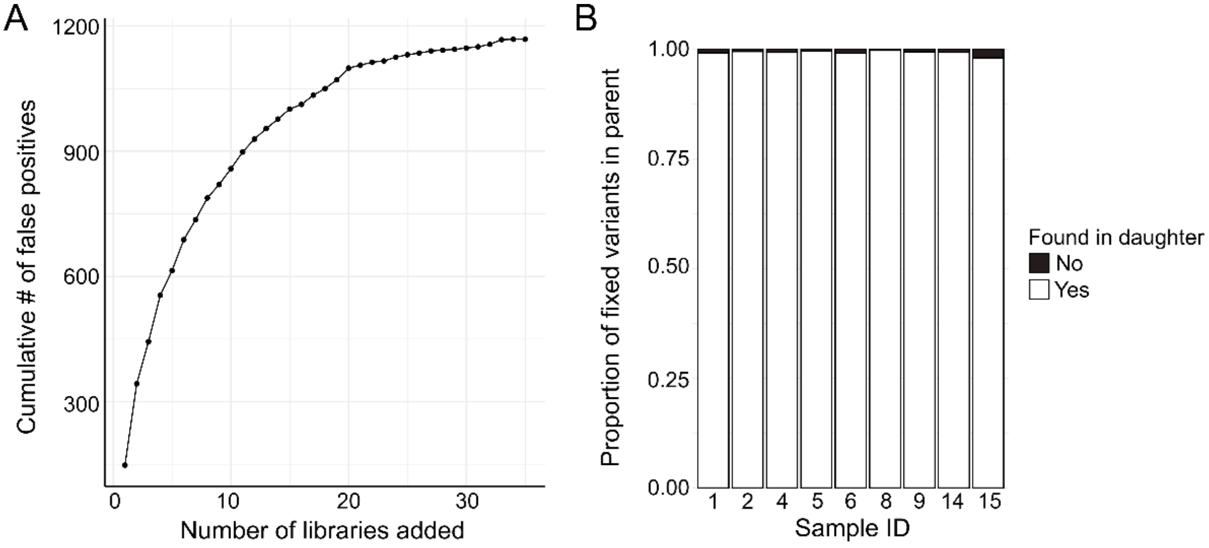

Figure S10. Identification of *de novo* mutations, related to Figure 4. A) The cumulative number of false positive *de novo* mutations decreases as the number of libraries increases. *De novo* mutations were identified in the consecutive head lines and then compared against unrelated datasets. This cross-referencing allowed us to eliminate most false positives—mutations that also appear in other datasets and are therefore unlikely to be true *de novo* mutations. B) False negative variant calling rates are low. By assessing the proportion of SNPs that are heterozygous in the parent worm (allele frequency of 0.5) but absent from the offspring, we estimate the false negative rates of variant calling in our dataset to be 0.008 ± 0.005 (mean ± standard deviation).

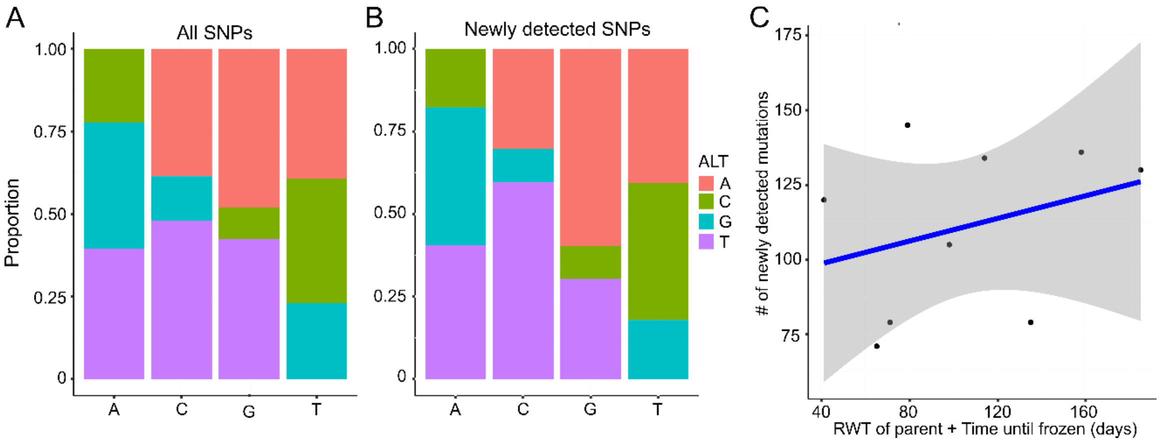

Figure S11. Characterization of putative *de novo* variants, related to Figure 4. A-B) Mutation patterns of A) all SNPs from the “head” dataset are broadly similar to B) those of the newly detected variants, suggesting that newly detected variants are unlikely to be driven by error. C) Number of newly detected mutations as a function of time. To consider all time where mutations could arise we add the reproductive waiting time (RWT) of the parent (time since the parent was born until division) and the age of the worm when frozen. Blue line shows a linear fit. Shaded area is the 95% confidence interval.

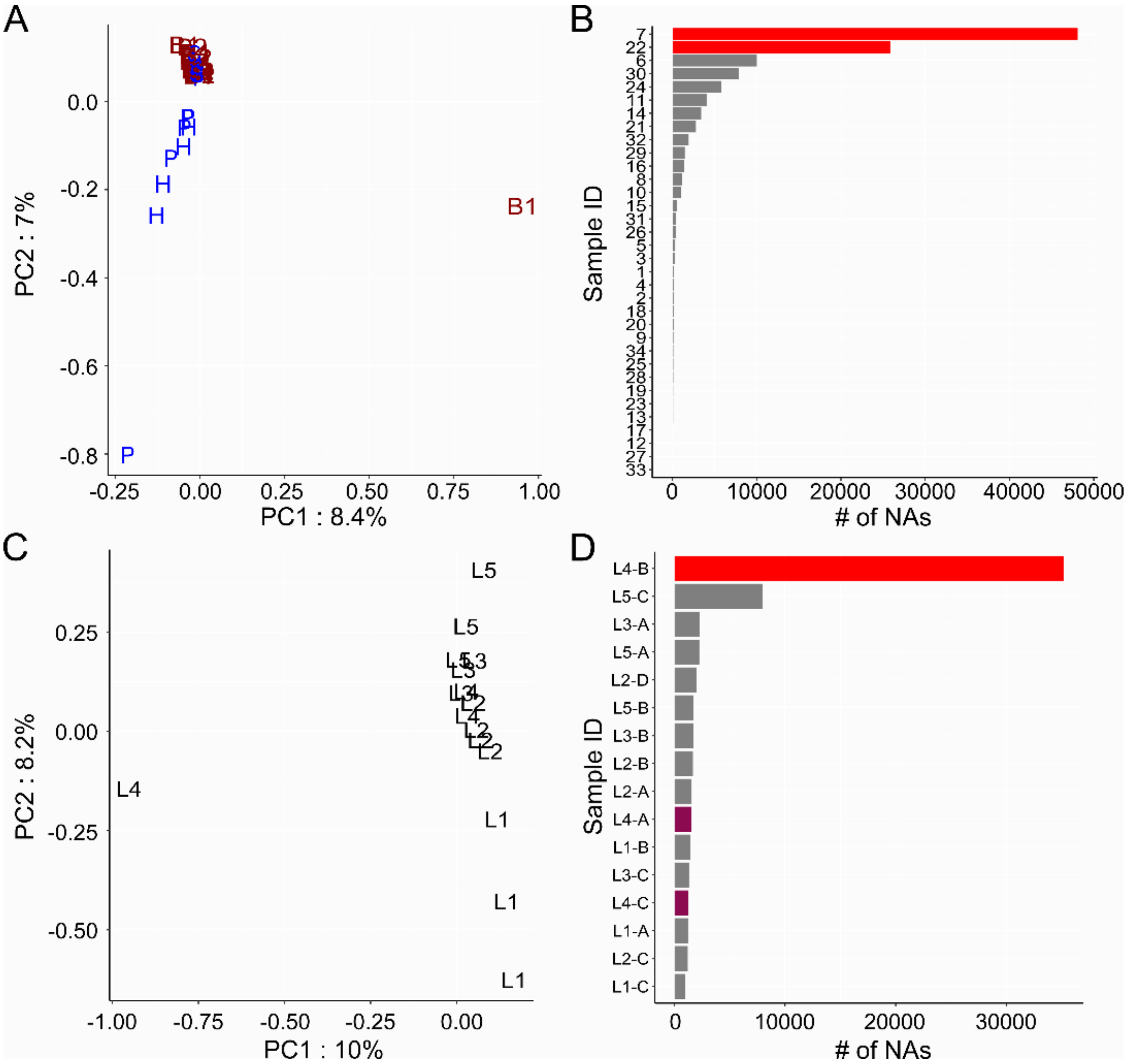

Figure S12. Outlier removal, related to STAR Methods. A) PCA of all non-perturbed and bottleneck sequenced samples. The letters indicate the lineages (P, H, F, S, B1, B2). Blue indicates the non-bottleneck lineages and red indicate the bottleneck lineages. One B1 and one P sample were outliers. B) The same outlier samples have high levels of missing genotyping data. C) PCA of head line data shows that one sample from lineage 4 (L4) is an outlier. D) The same outlier sample has a high number of missing genotype data. We removed all samples from the L4 lineage (magenta) since the detection of new mutations relies on other samples in the lineage.

Supplemental Tables

Table S1. Samples used to compare non-perturbed and bottleneck lineages. Related to STAR Methods and Figures 2-3.

Sample ID	Family	Generational history
21009D-02-01	Fritz	11111101101
21009D-02-02	Hans	
21009D-02-03	B1	
21009D-02-04	B2	
21009D-02-05	Fritz	01011111111
21009D-02-06	Hans	
**21009D-02-07**	**B1**	
21009D-02-08	B2	
21009D-02-09	Fritz	01110111110
21009D-02-10	Hans	
21009D-02-11	B1	
21009D-02-12	B2	
21009D-02-13	Fritz	11111111001
21009D-02-14	Hans	
21009D-02-15	B1	
21009D-02-16	B2	
21009D-02-17	Sophie	0011111101
21009D-02-18	Peter	
21009D-02-19	B1	
21009D-02-20	B2	
21009D-02-21	Sophie	1101101101
**21009D-02-22**	**Peter**	
21009D-02-23	B1	
21009D-02-24	B2	
21009D-02-25	Sophie	0011111110
21009D-02-26	Peter	
21009D-02-27	B1	
21009D-02-28	B2	
21009D-02-29	Sophie	0011111011
21009D-02-30	Peter	
21009D-02-31	B1	
21009D-02-32	B2	
21009D-02-33	Sophie	01110111110
21009D-02-34	Peter	

Generational history refers to the life history of that sample where 1 indicates a head offspring and 0 indicates tail offspring (see Figure S1). Bold samples were removed as outliers.

Table S2. Samples used in the “Heads” Dataset. Related to STAR Methods and Figure 4.

Sample number	Lineage-Generational ID	Generational history
1	1-A	11111011001
2	1-B	111110110001
3	1-C	11111011000001
4	2-A	11010111101
5	2-B	110101111001
6	2-C	1101011110001
7	2-D	110101111000001
8	3-A	1111010101
9	3-B	11110101001
10	3-C	111101010001
**11**	**4-A**	**11101111111111111101**
**12**	**4-B**	**111011111111111111001**
**13**	**4-C**	**1110111111111111110001**
14	5-A	1000111001
15	5-B	0001110001
16	5-C	00011100001

Generational history refers to the life history of that sample where 1 indicates a head offspring and 0 indicates tail offspring (see Figure S1). Bold samples were removed as outliers.

Table S3. Genes impacted by substrain-specific indels in PH and FS. Related to Figure 2. Blast was performed in planmine.

Gene	Strain	TOP-BLAST hit	Summary	Frameshift impacting domain	Notes
MSTRG.4899.3 - SMEST013737001.1	PH	Mus musculus - dynein, axonemal, heavy chain 3	Dynein	NO	
SMEST003729001.1	PH	Harpegnathos saltator - piggyBac transposable element-derived protein 4-like	TE	YES	
MSTRG.1962.5 - SMEST003567001.1	PH	Homo sapiens - patched 1	Patched like protein	YES	
SMEST015949002.1	PH	Tribolium castaneum - uncharacterized LOC103313431	Uncharacterised - DNApol domain	NO	No domains detected in interpro
SMEST035238001.1	PH	NULL	Uncharacterised	NA	
SMEST043489001.1	PH	Drosophila melanogaster - Ral interacting protein	RalA binding protein	NO	
SMEST043490001.1	PH	Schistosoma haematobium - hypothetical protein	Potentially extracellular matrix glycoprotein	YES	
SMEST062835001.1	PH	Homo sapiens - TNF receptor associated factor 3	TNF receptor	NO	No domains detected in interpro
SMEST062836001.1	PH	NULL	Uncharacterised	NA	
SMEST065771001.1	PH	NULL	Uncharacterised	NA	
SMEST072461001.1	FS	Tetranychus urticae - rho GTPase-activating protein 190-like	Rho GTPase	YES	

Supplemental References

S1. Thomas, M.A., and Schötz, E.-M. (2011). SAPling: a Scan-Add-Print barcoding database system to label and track asexual organisms. The Journal of experimental biology *214*, 3518–3523. https://doi.org/10.1242/jeb.059048.

S2. Carter, J.A., Lind, C.H., Truong, M.P., and Collins, E.-M.S. (2015). To each his own. Journal of Statistical Physics *161*, 250–272. https://doi.org/10.1007/s10955-015-1310-1.

S3. Dunkel, J., Talbot, J., and Schötz, E.-M. (2011). Memory and obesity affect the population dynamics of asexual freshwater planarians. Physical biology *8*, 026003. https://doi.org/10.1088/1478-3975/8/2/026003.

S4. Thomas, M.A., Quinodoz, S., and Schötz, E.M. (2012). Size Matters!: Birth Size and a Size-Independent Stochastic Term Determine Asexual Reproduction Dynamics in Freshwater Planarians. Journal of Statistical Physics *148*, 663–675. https://doi.org/10.1007/s10955-012-0514-x.

S5. Quinodoz, S., Thomas, M.A., Dunkel, J., and Schötz, E.M. (2011). The More the Merrier?: Entropy and Statistics of Asexual Reproduction in Freshwater Planarians. Journal of Statistical Physics *142*, 1324–1336. https://doi.org/10.1007/S10955-011-0157-3/METRICS.

S6. Guo, L., Bloom, J.S., Dols-Serrate, D., Boocock, J., Ben-David, E., Schubert, O.T., Kozuma, K., Ho, K., Warda, E., Chui, C., et al. (2022). Island-specific evolution of a sex-primed autosome in a sexual planarian. Nature *606*, 329–334. https://doi.org/10.1038/s41586-022-04757-3.
